# The Effects of Chain Length and NaCl Concentration on Phase Separation and Morphology of Polylysine Complexes with tRNA and dsDNA

**DOI:** 10.1021/acsomega.6c00154

**Published:** 2026-05-05

**Authors:** Kimiasadat Mirlohi, Kavya Famolari, Whitney C. Blocher McTigue

**Affiliations:** 1 Department of Chemical and Biomolecular Engineering, 1687 Lehigh University, Bethlehem, Pennsylvania 18015, United States; 2 Integrated Engineering and Arts and Sciences program (IDEAS), 1687 Lehigh University, Bethlehem, Pennsylvania 18015, United States

## Abstract

Electrostatic complexation between cationic polymers and nucleic acids underlies both fundamental biomolecular assemblies and emerging therapeutic technologies. Among these systems, polylysine/nucleic acid complexes provide a simple yet powerful model for probing how the molecular architecture and chain length dictate phase separation and salt-induced interactions. Despite extensive but separate studies on nucleic acid complexes and the effects of polymer length, the influence of polylysine chain length on phase behavior and morphology across different nucleic acids remains limited. Here, we systematically investigate complexes formed between poly-l-lysine (PLK) of defined lengths (30–800 residues) and three nucleic acid systems: baker’s yeast tRNA (75–80 bp) and two length ranges of salmon sperm dsDNA (200–500 and ≤2000 bp). Using turbidity assays and optical microscopy, we examined phase separation and morphological transitions across a broad NaCl concentration range (0–1500 mM) and compared them to previous work. Our results show that the presence of single- and double-stranded nucleic acids strongly influences polymer complex stability and morphology in the presence of salt. The salt resistance was strongly influenced by the shortest polymer in the complex, though the length of the longer partner showed only subtle shifts in this critical salt value. The nucleic acid type further modulated outcomes: in the absence of added salt, dsDNA at all length ranges exclusively formed precipitates, whereas tRNA consistently formed coacervate droplets with all PLK variants. Interestingly, nucleic acid chain lengths below 100 showed phase transitions in the presence of salt before transitioning to a single-phase solution, whereas the longer dsDNA chains did not show a transition. However, dsDNA consistently transitioned from precipitates to coacervates, while tRNA transitioned from coacervates to precipitates. We note that even with systems of two short polymers, complexes remained phase-separated well beyond physiological ionic strength. Together, these findings further establish that the chain length and nucleic acid architecture are key determinants of polyelectrolyte complex morphology and stability across a range of ionic strengths. By linking molecular features to macroscopic behavior, this work provides design principles for engineering nucleic acid–polymer assemblies with tunable properties for biomaterials and therapeutic applications.

## Graphical abstract

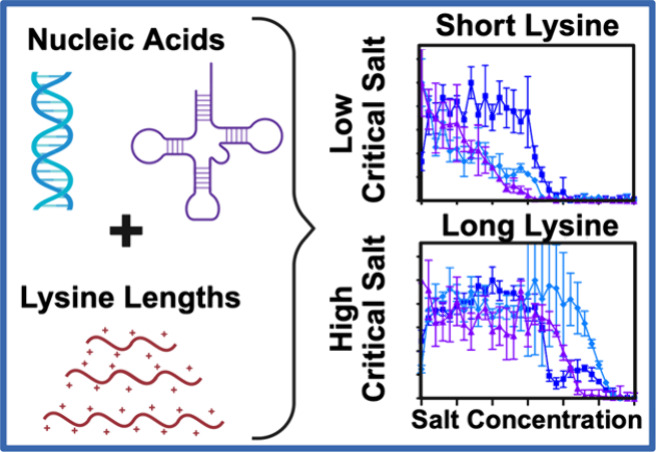

## 1 Introduction

Electrostatic interactions between oppositely charged macromolecules drive a wide range of biological assemblies, from ribonucleoprotein granules^1^ to viral capsids.^2^ In vitro, these interactions are often studied through polyelectrolyte complexation, in which cationic and anionic polymers undergo associative phase separation.^3^ Such systems not only provide fundamental insights into soft matter physics but also serve as platforms for developing nucleic acid delivery vehicles,^4^ protein encapsulation strategies,^5^ and biomaterials with tunable stability.^6^ Among cationic polymers, poly-l-lysine (PLK) has been extensively used as a model system due to its defined primary structure, synthetic accessibility, and broad relevance in biomedical applications.^7^ Its interactions with nucleic acids, including dsDNA and tRNA, capture key features of charge-driven assembly in both natural and engineered contexts.^8,9^

The length of the polycation chain is a critical parameter in determining complex formation and stability. Longer chains introduce more charge per molecule, increasing multivalency and promoting bridging interactions across multiple nucleic acid strands.^10^ This enhanced connectivity can stabilize complexes and influence whether phase separation occurs^11^ and the size of the two-phase region.^12–14^ Equally important, however, is the relative length of the two interacting polyelectrolytes.^15^ A large disparity in chain length between the polycation and nucleic acid can promote uneven charge compensation, steric mismatches, or asymmetric bridging interactions,^16^ all of which may bias complexes toward inconsistent morphologies. In contrast, when the sizes of the interacting species are more closely matched, the likelihood of balanced charge pairing and dynamic rearrangement increases, favoring more stable phase separation.^16,17^ Despite its clear importance, the role of chain length disparity—whether the polycation is much shorter or much longer than the nucleic acid—has received limited systematic attention in nucleic acid–polylysine complexes. While these principles suggest that chain length, both in absolute terms and relative to the complexing partner, should strongly govern phase behavior and morphology, systematic studies directly testing this relationship with nucleic acids and polycations of broad sizes remain scarce. The structure of the nucleic acid also strongly shapes complex morphology. dsDNA and tRNA exhibit distinct nucleic acid signals in circular dichroism analysis (Figure S1), as dsDNA has a double-helical structure with fewer single-chain sections and a continuous charge distribution, which limits rearrangement and often biases assemblies toward aggregated or precipitated states.^18^ Though short dsDNA has been shown to form liquid crystal coacervates when complexed with polylysine,^19^ this liquid crystal behavior could be suppressed by introducing dangling ends into the DNA. tRNA, by contrast, is compact and highly folded, with hydration pockets embedded within its cloverleaf architecture, which includes single-chain sections; these characteristics enable more flexible, dynamic interactions with PLK and tend to favor the formation of coacervate droplets over precipitation. Interestingly, shorter nucleotides, such as ATP,^20,21^ a precursor to both DNA and RNA, ADP, and AMP, tend toward coacervation, further suggesting that length is a key parameter.^22^

Another key determinant of polyelectrolyte complex stability is ionic strength. Physiological environments contain salts at concentrations that can screen electrostatic interactions and destabilize complexes. Salt resistance is therefore not only a window into the fundamental physics of polyelectrolyte assemblies but also a practical measure of their viability in biomedical formulations. Beyond stability, ionic strength can also be leveraged as a tunable parameter to control function. For example, complexes remain intact under extracellular ionic conditions, commonly modeled as ∼150 mM NaCl,^23^ but selectively dissolve at higher salt concentrations and act as responsive carriers, releasing their cargo only after reaching specific ionic strength targets. Previous studies have explored the phase behavior of nucleic acid and polylysine complexes that are close in length in the presence of upward of 1 M NaCl and have also observed shifts in the phase and shape of the droplets and precipitates.^24^ Similarly, designing assemblies with defined critical salt thresholds provides a strategy for on-demand disassembly under saline conditions, aquatic applications, and therapeutic formulation and delivery. Previous work has shown that nucleic acid–polycation complexes exhibit shifts in morphology with varying salt concentrations and minor changes in polymer length,^24^ but morphological changes at higher salt concentrations and the extent to which larger gaps between the polycation chain length and nucleic acid size alter this behavior remain unresolved.

Here, we address this gap by expanding on work by the Tirrell group^24,25^ and others,^26,27^ systematically varying the PLK chain length and nucleic acid type and size to probe their combined effects on phase separation and morphology under increasing NaCl concentrations, specifically at longer PLK chain lengths and larger nucleic acid sizes. We compare complexes formed between PLK polymers of defined lengths (30–800 residues) with baker’s yeast tRNA (75–80 bp) and salmon sperm dsDNA (200–500 bp and ≤2000 bp). Using turbidity measurements and optical microscopy, we map critical salt concentrations (CSCs) and morphological outcomes across a broad ionic strength range (0–1500 mM).

We hypothesized that the PLK chain length would correlate with increased salt resistance and more persistent phase separation, whereas the nucleic acid architecture would dictate whether complexes favor liquid-like coacervation or solid precipitation. Specifically, we expected tRNA to preferentially form coacervates across PLK lengths, whereas dsDNA, due to its extended, rigid structure, would be biased toward precipitation. Furthermore, we posited that the more mismatched systems will have salt resistances more heavily influenced by the shorter partner.^12^ By linking molecular features such as chain length and nucleic acid size to emergent phase behavior, this study provides further insights into the design of nucleic acid–polymer assemblies with tunable stability and morphology.

## 2 Materials and Methods

### 2.1 Materials

Poly-l-lysine hydrobromide (PLKBr) with defined chain lengths of 30, 50, 100, 250, 400, and 800 residues was purchased from Alamanda Polymers, Inc. (Huntsville, AL) and used without further purification. Baker’s yeast tRNA (tRNA, 75–80 bp) and salmon sperm double-stranded DNA (dsDNA, 200–500 and ≤2000 bp) were obtained from Thermo Fisher Scientific. Stock solutions of 10 mM polylysine (on a monomer basis) were prepared gravimetrically in deionized water and adjusted to pH 7.0 (±0.03 pH units) using a Mettler Toledo InLab Expert Pro-ISM pH electrode. Small volumes of 1 or 0.1 M HCl and 1 or 0.1 M NaOH were added as needed for adjustment. A 0.5 M 2-(4-(2-hydroxyethyl)-1-piperazinyl)-ethanesulfonic acid (HEPES) buffer (Thermo Fisher Scientific) was prepared at pH 7.0 (±0.03) and used in all experiments. Sodium chloride (NaCl) was purchased from Thermo Fisher Scientific, and stock solutions were prepared at 2 M for salt resistance studies.

### 2.2 Sample Preparation, Turbidity Measurements, and Optical Microscopy

Coacervate samples were prepared by first pipetting ultrapure water (Aries FilterWorks, 18.2 MW cm) and HEPES buffer into 1.5 mL microcentrifuge tubes (Thermo Fisher Scientific), followed by vortexing for 10 s to ensure thorough mixing. Nucleic acids (tRNA or dsDNA) were then added to the tube and vortexed, after which poly-l-lysine (PLK) of the desired chain length was introduced. The mixture was vortexed immediately to promote fast and complete mixing. A typical experiment contained a total polymer concentration of 2 mM (on a monomer basis) and a constant concentration of 10 mM HEPES buffer, with a sample volume of 120 μL, of which 115 μL was used for turbidity analysis. The details of the charge fraction calculations are provided in the SI (eq S3).

For salt resistance studies, samples were prepared using the same procedure, with NaCl added to achieve final concentrations ranging from 0 to 1500 mM across a 30-sample series. A 31st sample consisting of only water and buffer was prepared as a blank for each experiment. All experiments were carried out in triplicate.

Following preparation, three 35 μL aliquots of each sample were transferred into a 384-well plate (Falcon) for turbidity measurements. Absorbance was measured using a BioTek Cytation 5 microplate reader at 562 nm, with turbidity defined as −ln­(*I*/*I*_0_), where *I*_0_ is the incident light intensity and *I* is the transmitted intensity. Measurements were referenced against the wells containing blank samples of water and buffer. Samples were then imaged by optical microscopy (Rebel Microscope, Echo) to verify the presence or absence of phase separation and to assess droplet or precipitate morphology.

## 3 Results and Discussion

### 3.1 Baseline Turbidity and Phase Behavior in the Absence of Salt

At 0 mM NaCl, all nucleic acid–polymer systems peaked in turbidity near 0.50 charge fraction (+) (mol/mol) (Figure S2). Additionally, the turbidity curves were organized based on polylysine length for all nucleic acids to demonstrate the influence of the nucleic acid type and length on the turbidity behavior of each system at the same polycation length (Figure 1). Both PLK_30_ and PLK_50_ are shorter than tRNA and are close in length to tRNA, and they exhibit the narrowest turbidity peaks. A similar pattern is seen with PLK_250_ and PLK_400_ in complexation with 200–500 bp dsDNA. This pattern is more difficult to consistently observe with ≤2000 bp dsDNA due to its broader length distribution and higher polydispersity. Another noteworthy observation was made with the raw, non-normalized turbidity measurements: The shape of the curve and the magnitude of the turbidity readings almost always overlapped for both dsDNA lengths, while the magnitude of the peak was much higher for tRNA systems with a curve shape distinctively different from those of the dsDNA variants for each respective PLK length (Figure S3). The consistently higher turbidity readings for tRNA systems can be explained by the way coacervate droplets interact with light compared to solid precipitates, and this observation further confirms the defining role nucleic acid structure plays in complexation behavior, as well as its explicit effect on the results obtained through analysis tools commonly used in the field, such as turbidity measurements.

**Figure 1:**
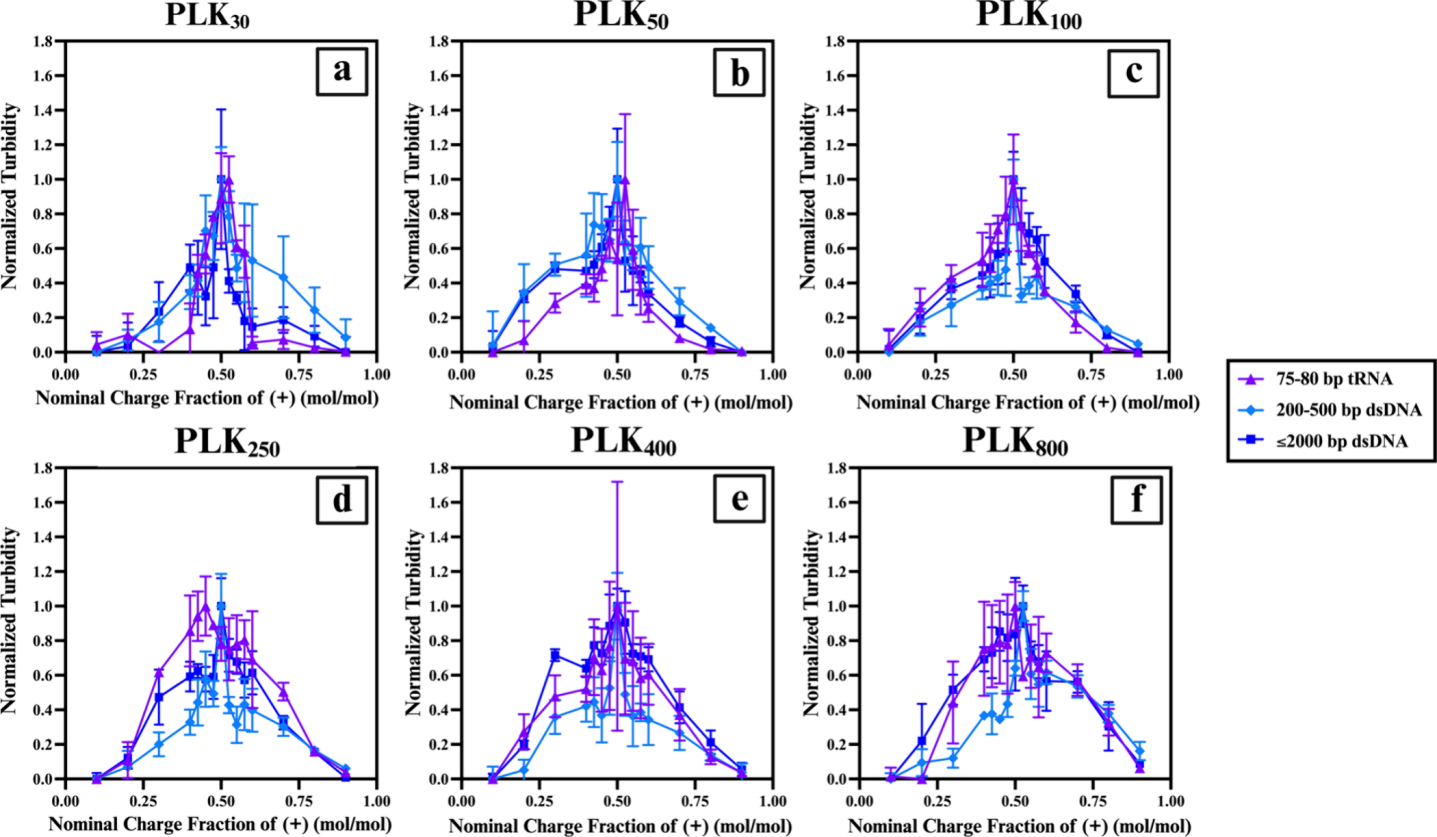
Normalized turbidity versus nominal charge fraction of (+) (mol/mol) for all nucleic acid types for each polymer length, highlighting the effects of the nucleic acid type and structure. tRNA is marked with purple triangles, 200–500 bp dsDNA is marked with light blue diamonds, and ≤2000 bp dsDNA is marked with dark blue squares. (a) PLK_30_ complexes, (b) PLK_50_ complexes, (c) PLK_100_ complexes, (d) PLK_250_ complexes, (e) PLK_400_ complexes, and (f) PLK_800_ complexes.

To better visualize the effects of polymer length, the turbidity curves were organized based on the nucleic acid type (Figure S4). The overall pattern across all these curves is that, as the polycation length increases, high turbidity readings become possible over a wider range of charge fractions, leading to a visually broader turbidity curve. This can be explained by the increase in the possibility and probability of inter- and intramolecular electrostatic interactions (e.g., the attraction between PLK side chains and the backbone of the nucleic acids) and interference (e.g., the repulsion between the longer nucleic acid chains with themselves). Additionally, longer polyelectrolytes allow bridging between chains, which can lead to more interactions and complexation, even when the charges are not near balance at 0.50 charge fraction.

All PLK-tRNA mixtures formed liquid coacervate droplets across the full PLK length series (30–800 residues) at 0 mM NaCl (Figure 3a), whereas all PLK-dsDNA systems (both 200–500 and ≤2000 bp) produced solid-like precipitates rather than droplets (Figure 2b,c). These qualitative outcomes were consistent across triplicates and were confirmed by turbidity and brightfield microscopy immediately after mixing. The tRNA results indicate a robust liquid–liquid phase separation (LLPS) window with both short and long polycation lengths (e.g., PLK_30_ and PLK_800_), whereas the dsDNA systems show a bias toward solid complexation at the same polymer concentrations and pH. Together, these baseline observations already point to the nucleic acid architecture as a determinant of the material state at a given charge stoichiometry.

**Figure 2:**
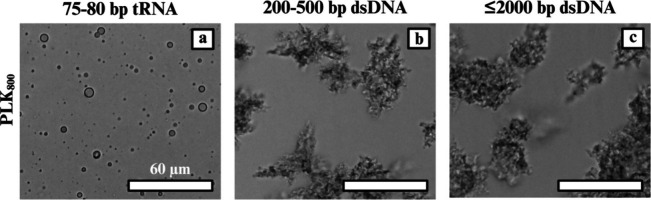
Representative micrographs of nucleic acid complexes are shown only with PLK_800_. With all polylysine lengths, (a) tRNA forms coacervate droplets, whereas (b) 200–500 dsDNA and (c) ≤2000 bp dsDNA form precipitates with all polylysine lengths.

Mechanistically, the preference of dsDNA for precipitated complexes aligns with prior observations for double-stranded and rigid nucleic acids^24^ and the fact that increasing linear charge density and chain length can favor solid complexes over coacervates, particularly when the polyelectrolytes are length-mismatched and when interchain packing and bridging dominate over dynamic rearrangement.^16^ Foundational studies and reviews attribute this to counterion release-driven binding coupled with limited chain mobility and higher interfacial tension, which together push assemblies toward kinetically trapped, solid morphologies in certain sequence and length regimes.^14,28^

### 3.2 Salt Resistance Depends on Both the PLK Length and Nucleic Acid Size and Structure

To understand the phase behavior as salt increases, we selected a single polymer concentration (on a monomer basis, Figure 3 dashed gray line) and used it as a reference across all our systems, rather than constructing an entire phase diagram (Figure 3). We studied stability as a function of ionic strength by incrementally increasing NaCl from 0 to 1500 mM in distinct samples containing equal concentrations of PLK and nucleic acid on a monomer basis (charge fraction, *f*_+_, of 0.5), and identifying the CSC, the salt concentration at which phase separation was no longer visible, sometimes referred to as the salt resistance. Turbidity measurements versus NaCl concentration are shown in Figure S5, indicating a similar trend across most systems: as the salt concentration increases, complexes dissolve back into solution due to charge screening, leading to a decrease in turbidity. It is possible that the longer PLK length and the bias toward dsDNA-forming precipitates have led to stronger interactions and, thus, prevented the formation of a single-phase system over the range of salt concentrations explored (dsDNA-2000 with PLK_800_, Figures S16 and S17).

**Figure 3:**
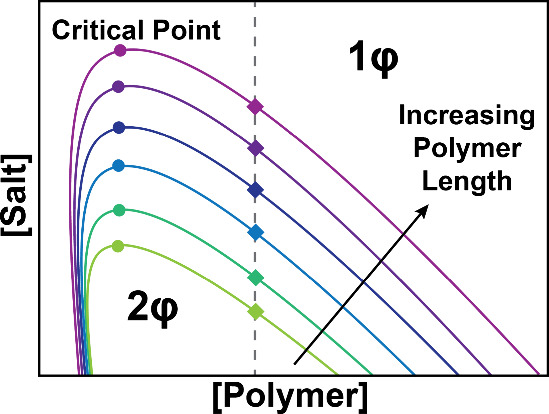
Schematic of a phase diagram as a function of increasing polymer length. As the polymer length increases, the area under the binodal curve increases. Each color represents a different length, with the colored circles indicating the critical point and the diamonds representing the system’s salt resistance, or critical salt concentration, at a constant polymer concentration (dashed gray line). Under the curve is the two-phase region, with the region above the binodal curve as the single-phase region.

To better visualize the effects of the nucleic acid type, normalized turbidity measurements versus salt concentration were organized by polymer length (Figure 4). We observed a consistent pattern: tRNA systems often had the lowest turbidity readings across the salt addition range, while the ≤2000 bp dsDNA systems had the highest. This aligns with the expectation that longer, more rigid nucleic acids will exhibit greater resistance and be more consistently resilient to salt addition. We also organized the data by nucleic acid type to highlight the effects of polymer length, and this analysis shows that while PLK_30_ and PLK_50_ had the lowest turbidity measurements throughout, PLK_400_ and PLK_800_ consistently showed the greatest resistance to salt addition (Figures S6 and S7).

**Figure 4:**
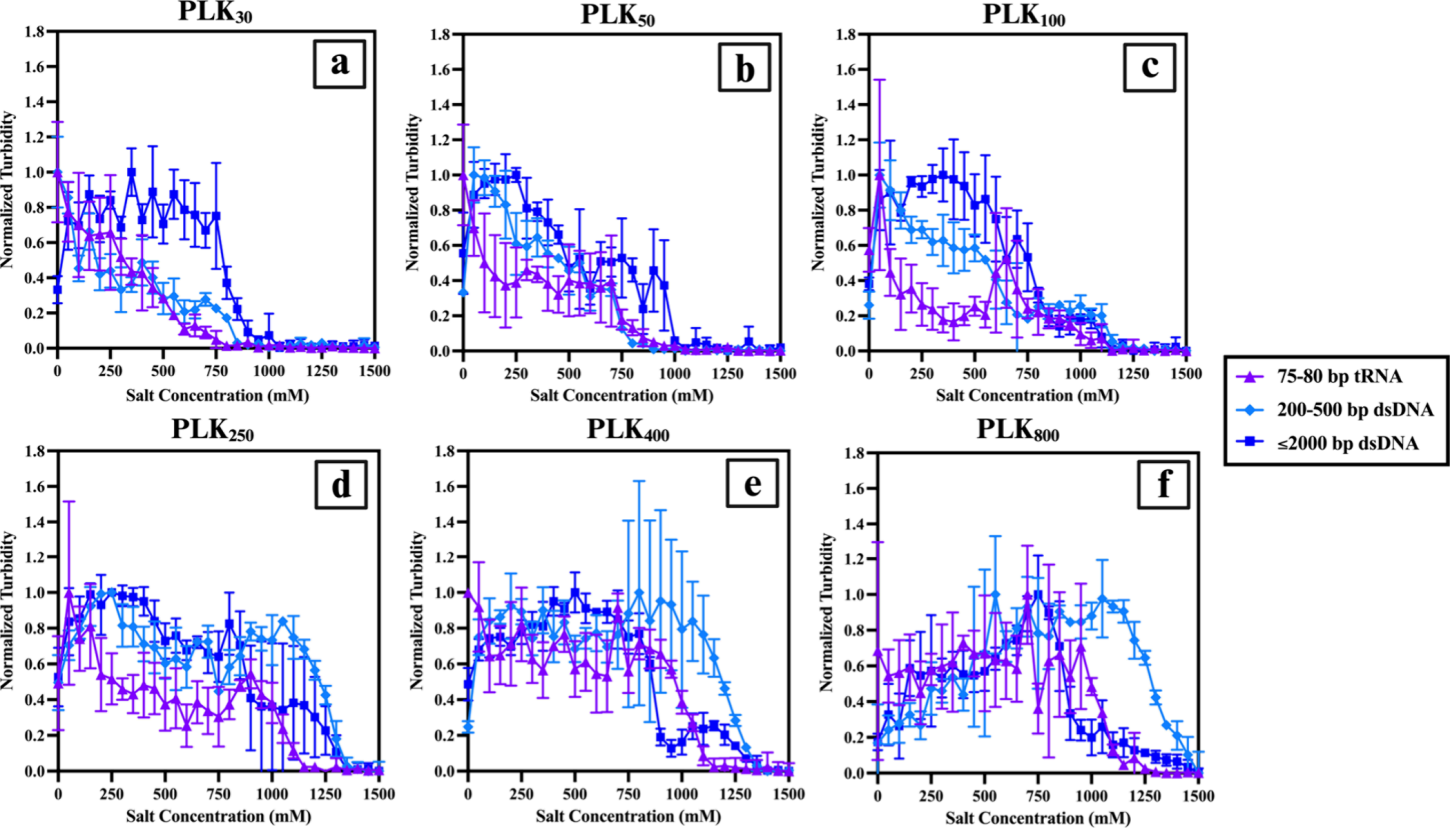
Normalized turbidity versus salt concentration (mM) for all nucleic acid types for each polymer length, highlighting the effects of the nucleic acid type and structure at *f*_+_ = 0.5. tRNA is marked with purple triangles, 200–500 bp dsDNA is marked with light blue diamonds, and ≤2000 bp dsDNA is marked with dark blue squares. (a) PLK_30_ complexes, (b) PLK_50_ complexes, (c) PLK_100_ complexes, (d) PLK_250_ complexes, (e) PLK_400_ complexes, and (f) PLK_800_ complexes.

For dsDNA, the CSC increased with PLK length (Figures S7 and S8), while it was the shorter polymer and then showed less dramatic changes. Complexes with PLK_30_ and PLK_50_ had visibly lower CSC values, ranging from 850 to 1000 mM, with the longer dsDNA. Complexes with PLK_100_ typically remained phase-separated at up to 1100–1150 mM added salt, while PLK_250_ and PLK_400_ showed higher CSC at up to 1300 mM. We did not observe a salt limit within the 0–1500 mM NaCl range for the PLK_800_ complexed with the ≤2000 bp dsDNA system. The CSC values and the salt concentrations at which morphological changes were observed are summarized in Table 1 and Figure 5 as a function of the smallest polymer length for all systems. It is also noteworthy that, while previous work has highlighted that the phase behavior of mismatched coacervates is highly dependent on the shortest chain,^12,17^ here, we have highlighted the effect of varying the cationic polymer length at a constant anionic chain length, and the role it plays in phase separation and complexation morphology.

**Table 1:** Salt Resistance Points and the Salt Concentrations at which the Morphology of Complexes Transitions for Each System

change in morphology (mM), salt resistance (mM)						
nucleic acid type	PLK_30_	PLK_50_	PLK_100_	PLK_250_	PLK_400_	PLK_800_
75–80 bp tRNA	700	900, 950	1000, 1150	1050, 1150	1050, 1150	1050, 1150
200–500 bp dsDNA	850	850	1150	1300	1300	1500
≤2000 bp dsDNA	950	1000	1100	1300	1300	N/A

**Figure 5:**
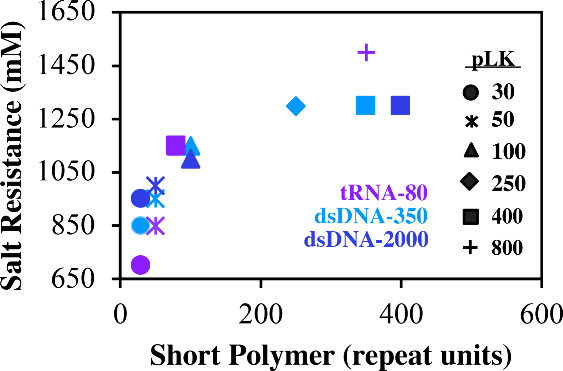
Salt (NaCl) resistance curves for tRNA (75–80 bp), dsDNA (200–500 bp, light blue), and dsDNA (≤2000 bp, dark blue) as a function of poly­(l-lysine) chain length (symbols). Note that, for calculations, the rounded averages for 75–80 bp tRNA and 200–500 bp dsDNA were used. The longest length (2000) was used for ≤2000 bp dsDNA.

If we expand our results to include those from Vieregg et al. (Table S3), who studied short dsDNA and ssDNA with four lengths of polylysine,^24^ we further observe two regions of salt resistance (Figure 6). As suggested by PLK_30_ (Figure 5, circles and stars), short polymers exhibit a sharp increase in the critical salt concentration. With longer polymer chains, we see a less dramatic increase in the salt resistance (Figure 6). Beyond using polylysine as the polycation, work in the literature (Figure S19) has shown similar trends as in Figures 5 and 7 with short peptides and poly­((vinylbenzyl) trimentylammonium),^25,27^ suggesting that these trends hold even across other polycations.

**Figure 6:**
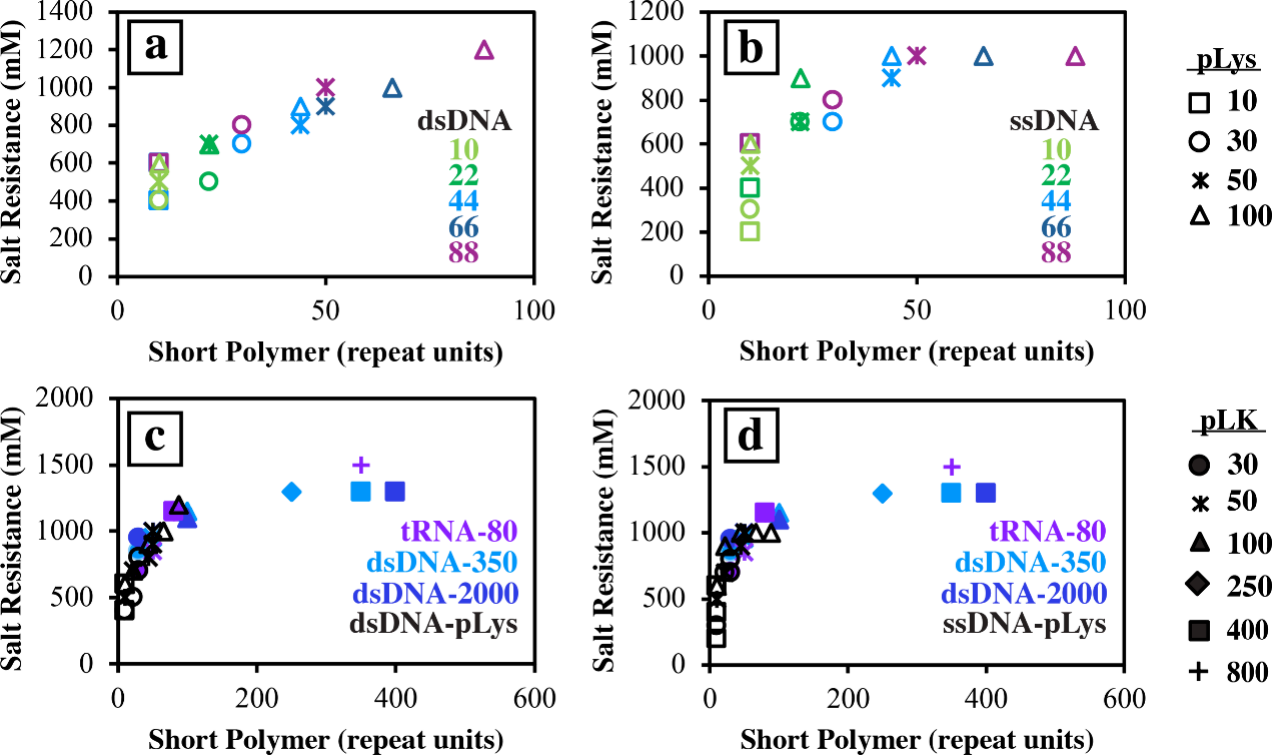
Salt (NaCl) resistance curves for short-length (10–88 nt) (a) dsDNA and (b) ssDNA complexed with varying lengths (10–100 aa) of polylysine from Vieregg et al.^24^ and compared to 75–80 bp tRNA (purple), 250–500 bp dsDNA (light blue), and ≤2000 bp dsDNA (dark blue) complexed with poly­(l-lysine) of lengths 30–800 aa in (c) and (d) from this study. In (c) and (d), the data from (a) and (b) have been colored black for ease of reading the graph. All data are tabulated in Table 1 and Table S3.

**Figure 7:**
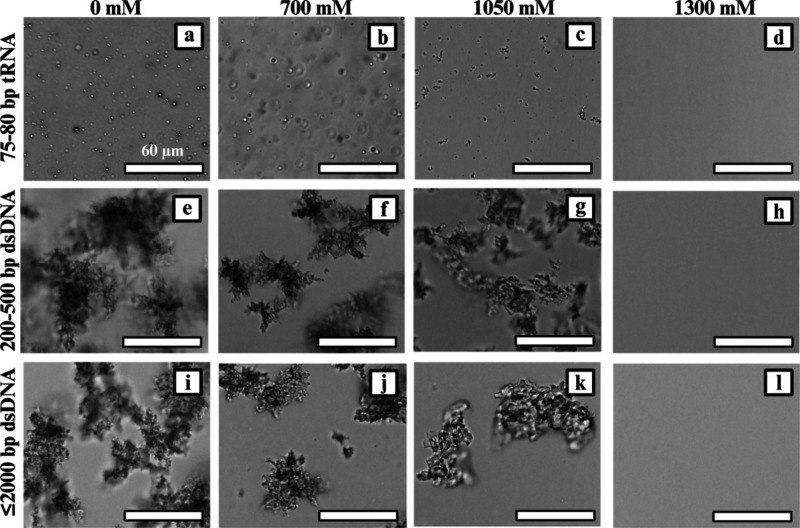
Micrographs of nucleic acids in complexation with PLK_250_ show changes in morphology as the concentration of added NaCl is increased. The shifts and transitions in the phase separation and morphology of PLK250 complexes with (a–d) 75–80 bp tRNA, (e–h) 200–500 bp dsDNA, and (i–l) ≤2000 bp dsDNA at 0, 700, 1050, and 1300 mM NaCl concentrations are shown. The complexes with PLK_250_ are representative of the general pattern observed, in which tRNA complexes lose their droplet shape as more salt is added, transitioning to solid-like aggregates before the solution returns to a single-phase state. On the other hand, complexes with dsDNA across both length ranges transition from precipitates to a single phase, without the clear phase transitions seen with tRNA systems.

To visually track qualitative shifts in morphology and phase separation with an increasing salt concentration, micrographs of representative systems with PLK_250_ and all three nucleic acid types are shown in Figure 7; the remaining systems are shown in Figures S9, S11, S13, S15, and S17. With the increase in NaCl concentration, tRNA complexes exhibited a clear liquid-to-single-phased transition: droplets gradually became less spherical (“melted”), transitioned into gel-like and solid-like aggregates based on visual characterization, and eventually disappeared at the CSC (Figures 7a–d and 8, purple data). A transitional behavior was also observed in the morphology of dsDNA complexes, shifting from precipitates with sharper, more distinct edges to softer, gel-like aggregates before the solution returned to a single-phase state (Figures 7e–l and 8, light and dark blue data), (Table 1. These observations reinforce the idea that phase separation is influenced by the interplay among chain flexibility and length, charge density, and size matching.

**Figure 8:**
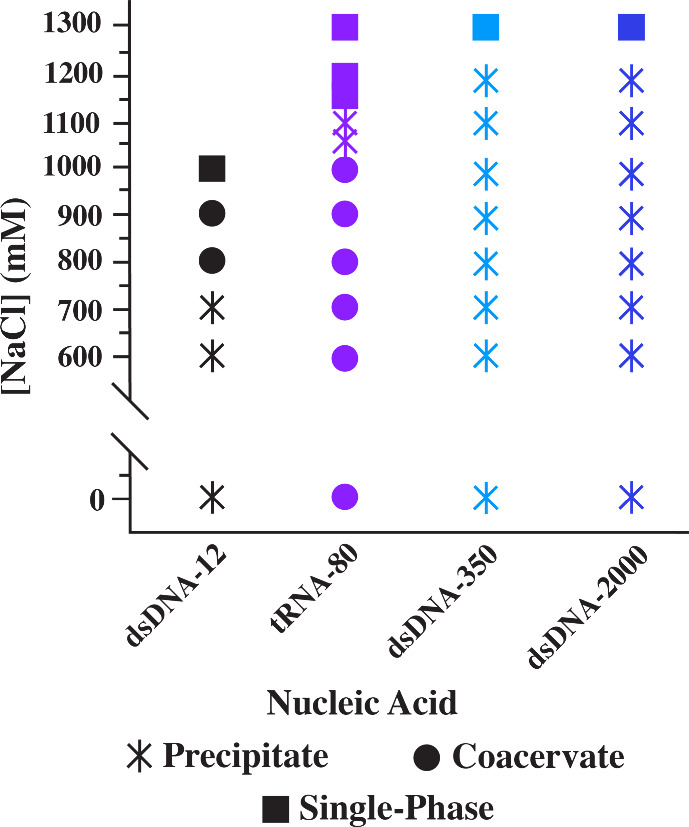
Phase diagram of a 12 nt dsDNA (black) from Shakya and King^26^ alongside 75–80 bp tRNA (purple), 250–500 bp dsDNA (light blue), and ≤2000 bp dsDNA (dark blue) when complexed with polylysine of length 240 (dsDNA-12) or 250 (all others). Stars represent precipitates, circles coacervates, and squares single-phase.

Interestingly, at shorter dsDNA lengths (Figure 8, black data^26^), we observe a transition opposite to tRNA, where the complexes start out as precipitates and relax more fully into coacervate droplets before becoming a single-phase solution. It is possible that the single- and double-stranded nature of tRNA rearranges as the salt concentration increases in the system. In ssDNA systems, complexes form as coacervates before transitioning to a single-phase solution.^24^ In the case of tRNA, the single-stranded portions may allow for coacervates at little or no salt. However, higher concentrations of the salt ions may promote chain rearrangement and allow more instances of double-strandedness through hydrogen bonds and base stacking,^29^ which likely promotes precipitation, before reaching a concentration at which it is no longer favorable for the polymers to complex. Additionally, this may account for the lack of a transition to coacervates with longer dsDNA strands, as higher salt concentrations are required to drive the system into a single-phase state, but these higher concentrations increase strand interactions. This increase in salt, which may induce overwinding of the dsDNA at these higher concentrations,^30^ may lead to the sharper transition between the precipitates and a single-phase solution. In shorter dsDNA, the higher salt concentrations potentially required for this phenomenon far surpass the critical salt concentration. Similar trends are observed for PLK chain lengths of 30, 50, 100, 400, and 800 (Figures S10, S12, S14, S16, and S18).

These length- and architecture-dependent CSC trends are consistent with classical and modern coacervation frameworks in which stability increases with multivalency (more charges per chain),^31^ stronger electrostatic correlations, and greater counterion-release entropic gains upon complexation, all of which are enhanced by longer polycations. Salt screens interpolymer attractions and reduces the free energy gain from counterion release; thus, systems with larger effective valency and tighter charge pairing maintain phase separation at higher ionic strengths.

### 3.3 Comparative Mechanistic Basis for the Behavior of tRNA and dsDNA Complexes

To rationalize the distinct phase outcomes observed for tRNA coacervate droplets and dsDNA solid precipitates under comparable conditions, specifically at little or no salt, we draw on established principles of polyelectrolyte complexation. The balance between dynamically reconfigurable charge pairing and longer-range bridging is governed primarily by (i) the degree of size matching between polyelectrolytes, (ii) effective multivalency and the entropy of counterion release, and (iii) backbone mechanics and local structure. Together, these factors shape the free energy that determines whether assemblies follow an LLPS regime or collapse into solid complexes as ionic strength increases. Here, we provide a qualitative analysis of our observations.

#### 3.3.1 Polymer Length, Backbone Mechanics, and Local Structure

As hinted previously, the length of the polymers strongly influences the salt stability of the polyelectrolyte complex. When one or both polymers are short, whether this is the nucleic acid or polylysine, the CSC is lower, sometimes considerably so, compared to longer polymer chain systems. For instance, if we consider tRNA across all PLK lengths, we observe a steady increase in the critical salt concentration when plotted against the shortest polymer length (Figure 5), but if we plot the same data now as a function of PLK length, we see a plateau in the salt resistance. In Figure S7, the curve for tRNA (purple) ceases to increase once a chain length of 100 is selected for PLK. This is the first instance where PLK is now the larger polymer, rather than tRNA. Furthermore, though subtle, there are smaller shifts based on the length of the longer polymer in terms of the CSC (Figure 6 and Figures S7 and S19), suggesting even more complex interactions between the polymers.

Additionally, increasing the PLK length raises the number of charges per chain and the number of potential contact points per encounter, enhancing the entropic gains from counterion release upon complexation. This increased effective valency strengthens electrostatic correlations and elevates salt resistance for both nucleic acids, consistent with the monotonic rise in CSC with PLK length observed while the nucleic acid is the larger polymer.

Lastly, the structural differences between dsDNA and tRNA play an important role in complexation. dsDNA’s secondary and tertiary structures in solution resemble a rigid rod, with the longer chains facilitating interstrand bridging and tight packing, stabilizing solid complexes at lower ionic strengths. By contrast, tRNA’s compact but more flexible cloverleaf structure allows for hydration pockets and supports rapid local rearrangements, while limiting long-range bridging, all of which are helpful characteristics for the formation of liquid coacervate droplets and eventual precipitate-like morphology at high salt.

## 4 Conclusions

This work establishes how the molecular length and nucleic acid structure collectively govern the phase behavior of poly-l-lysine coacervates. By systematically varying the PLK chain length and comparing tRNA and dsDNA, we show that both charge distribution and size matching strongly influence the stability and morphology of the resulting complexes. tRNA with all PLK chains favors liquid-like coacervates. In contrast, longer, more rigid nucleic acids, such as dsDNA, promote solid-like assemblies that are resistant to dissolution at higher ionic strength. Shorter DNA strands exhibit the same initial precipitate formation, followed by a transition to coacervates and finally to a single phase. Using flexible or irregular nucleic acid architectures or shorter DNA chains in low salt conditions supports liquid–liquid phase separation. In contrast, the extended, rigid backbones of dsDNA promote interchain bridging and the formation of dense, solid complexes, with longer chains forgoing any precipitate-to-coacervate transition at higher salt concentrations. Adjusting backbone stiffness, charge density, and ionic strength, therefore, enables control over the transition between liquid-like and solid-like morphologies. Together, these insights establish a molecular framework and a more predictive understanding of sequence, structure, and composition in engineering nucleic acid delivery vehicles and dynamic biomaterials.

## Supplementary Material



## Notes

Nucleic acid molecular weight, charge fraction, and normalization of turbidity calculations; circular dichroism analysis for nucleic acids; additional stoichiometry and salt study turbidity graphs; additional salt study micrographs; phase diagrams (PDF)

## Abbreviations

LLPS: liquid–liquid phase separation
PLK: poly-l-lysine
PLKBr: poly-l-lysine hydrobromide
HEPES: 2-(4-(2-hydroxyethyl)-1-piperazinyl)-ethanesulfonic acid
dsDNA: double-stranded DNA
tRNA: tRNA
CSC: critical salt concentration
