# Supplementary material for: The Effects of Chain Length and NaCl Concentration on Phase Separation and Morphology of Polylysine Complexes with tRNA and dsDNA
Source: ACS Omega. 2026 May 5;11(19):28334–41. doi: 10.1021/acsomega.6c00154 (PMC13191486; doi:10.1021/acsomega.6c00154)
Supplement: Supplementary file 1 [file ao6c00154_si_001.pdf]

Supplemental Information

The Effects of Chain Length and NaCl Concentration on Phase Separation and Morphology of Poly-Lysine Complexes with tRNA and dsDNA

Kimiasadat Mirlohi,[1] Kavya Famolari,[2] Whitney C. Blocher McTigue[1]*

[1]Department of Chemical and Biomolecular Engineering, Lehigh University, Bethlehem, PA 18015, US

[2]Department of Bioengineering, Lehigh University, Bethlehem, PA 18015, US

Nucleic Acid Molecular Weight Calculations

To estimate the molecular weights (MW) of the nucleic acids used in this study, we assumed an average base distribution of roughly 25% for A, U/T, G, and C.[1,2] This approximation is supported by sequence databases for *S. cerevisiae* tRNAs,[3,4] which show that despite sequence-level heterogeneity and post-transcriptional modifications, the average monomer composition converges near 330 g/mol per nucleotide (Table S1). These sources indicate that variations across individual sequences tend to average out at the population scale, making the 25% assumption both practical and sufficiently accurate for our model systems. Nucleic acid stocks (10 mg/mL) were obtained from Thermo Fisher Scientific, with nominal concentrations of ~30 mM on a nucleotide basis (Equations S1, S2, and S3). These samples were then diluted to 10 mM using sterile, ultrapure water for all experiments.

Our experimental observations support the validity of this MW estimation approach. Across all nucleic acid types examined, peak turbidity consistently appeared near a 0.5 charge fraction, indicating that the 10 mM nucleic acid solutions were electrostatically balanced with 10 mM polycation solutions under nominal 1:1 charge stoichiometry. This agreement between theoretical expectations and experimental outcomes confirms that our MW assumptions and derived concentrations were appropriate for the systems studied.

Supplemental Information

Table S1. Nucleotides and their respective molecular weights.[2]

Nucleotide	MW (g/mol)	NA Type
A	331.2	DNA/RNA
T	322.2	DNA
U	324.2	RNA
G	347.2	DNA/RNA
C	307.2	DNA/RNA

$$\boldsymbol{Average\ MW_{DNA} = \frac{331.2+322.2+347.2+307.2}{4} = 327.0 \approx 330\left(\frac{g}{mol}\right)} \quad \text{(Equation S1)}$$

$$\boldsymbol{Average\ MW_{RNA} = \frac{331.2+324.2+347.2+307.2}{4} = 327.5 \approx 330\left(\frac{g}{mol}\right)} \quad \text{(Equation S2)}$$

$$\boldsymbol{Concentration = \frac{0.010\left(\frac{g}{mL}\right)}{330\left(\frac{g}{mol}\right)} = 3.03 * 10^{-5}\left(\frac{mol}{mL}\right) \approx 30\ mM} \quad \text{(Equation S3)}$$

Supplemental Information

Charge Fraction Calculations

In this work, we define charge fraction (f_+) as the proportion of total charge contributed by the polycation, expressed on a monomer (charge) basis. Because all concentrations are reported per charge-bearing monomer, f_+ directly represents the relative contribution of positive to negative charges in a given mixture. Mathematically:

$$f_+ = \frac{[+]}{[+]+[-]} \qquad \text{(Equation S4)}$$

where [+] and [–] correspond to the molar concentrations of cationic polymer residues and anionic nucleic acid monomers, respectively.

From this definition:

- f_+ = 0.5 represents nominal charge neutrality (equal positive and negative charges).
- f_+ < 0.5 indicates an excess of nucleic acid.
- f_+ > 0.5 indicates an excess of polycation.

For example, a polymer concentration of 2 mM refers to 2 mM of total charge sites (not polymer chains), either cationic or anionic, depending on the polymer in question. Consistent with previous literature, the f_+ value at which maximum turbidity is observed (typically near 0.5) reflects the point of optimal charge compensation between the two components.

Supplemental Information

Normalization of Turbidity Calculations

To normalize the turbidity for each run, the maximum and minimum readings, after background subtraction, are used in equation S5, with the sample reading at each point set as x to then calculate the normalized value. This allows for the same range, 0 to 1, for each data set to observe any trends in shape.

$$x_{norm} = \frac{x - x_{min}}{x_{max} - x_{min}} \qquad \text{(Equation S5)}$$

Circular Dichroism Analysis for Nucleic Acids

Measurements were performed in 5 mm path-length quartz cuvettes (Starna Cells) using a Jasco J-815 circular dichroism spectrometer. Spectra were collected from 190 to 350 nm. The digital integration time was set to 1 s, and scans were acquired in step mode with a 1 nm bandwidth. Each sample was measured five times, and the spectra were averaged.

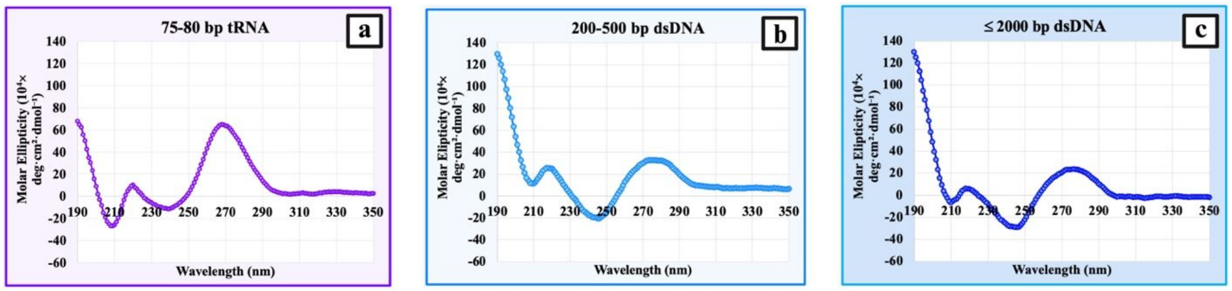

Figure S1. Circular dichroism analysis of all three nucleic acids, showing molar ellipticity ($10^4\times$ deg·cm²·dmol⁻¹) versus wavelength (nm). **(a)** CD analysis of tRNA, **(b)** CD analysis of 200-500 bp dsDNA, and **(c)** CD analysis of ≤2000 bp dsDNA.

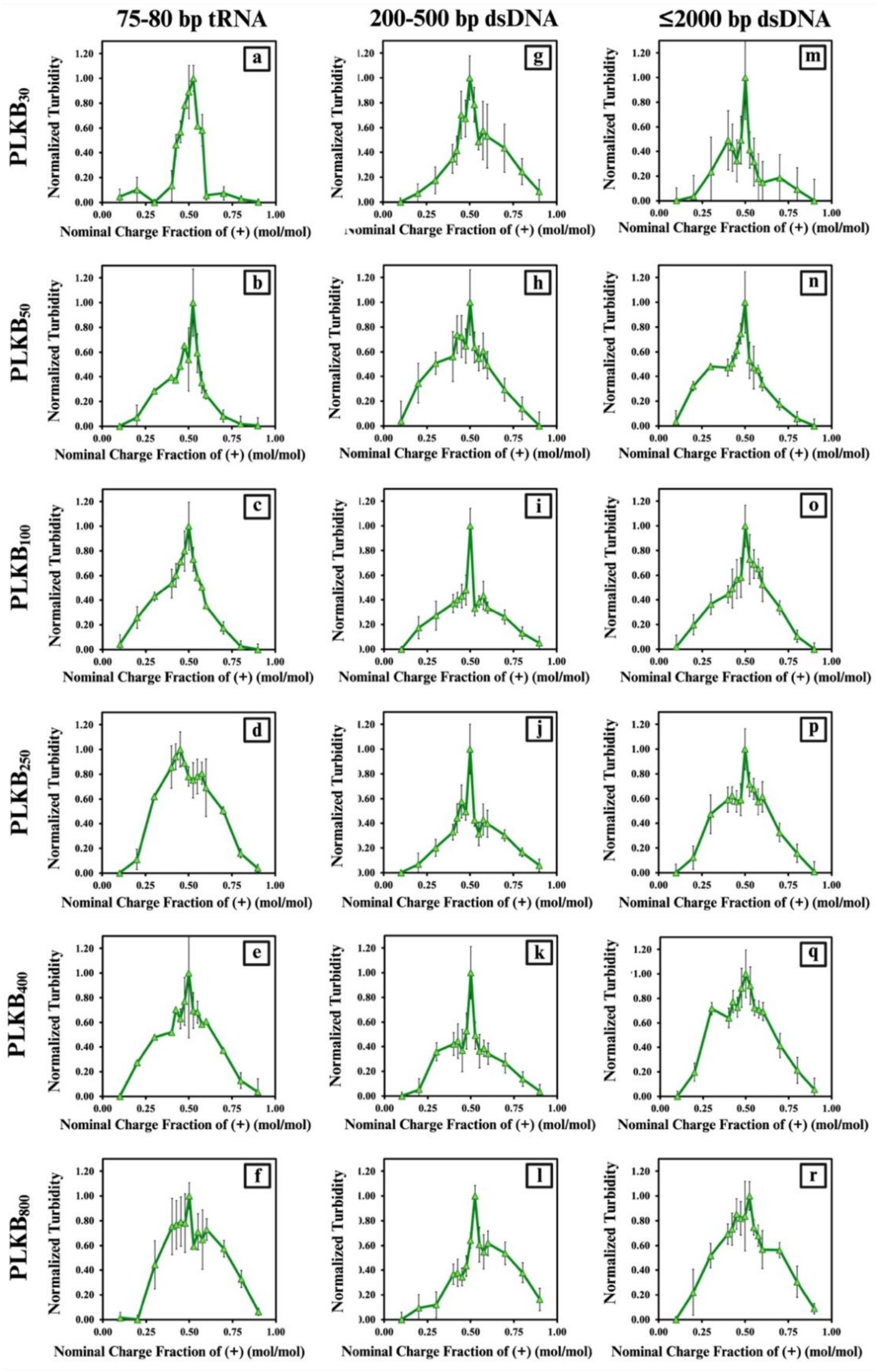

Figure S2. Normalized turbidity versus nominal charge fraction of (+) (mol/mol) for all systems. Normalized turbidity versus nominal charge fraction curves for all combinations of PLK_{30}, PLK_{50}, PLK_{100}, PLK_{250}, PLK_{400}, and PLK_{800} with **(a-f)** 75-80 bp tRNA, **(g-l)** 200-500 bp dsDNA, and **(m-r)** ≤2000 bp dsDNA are shown.

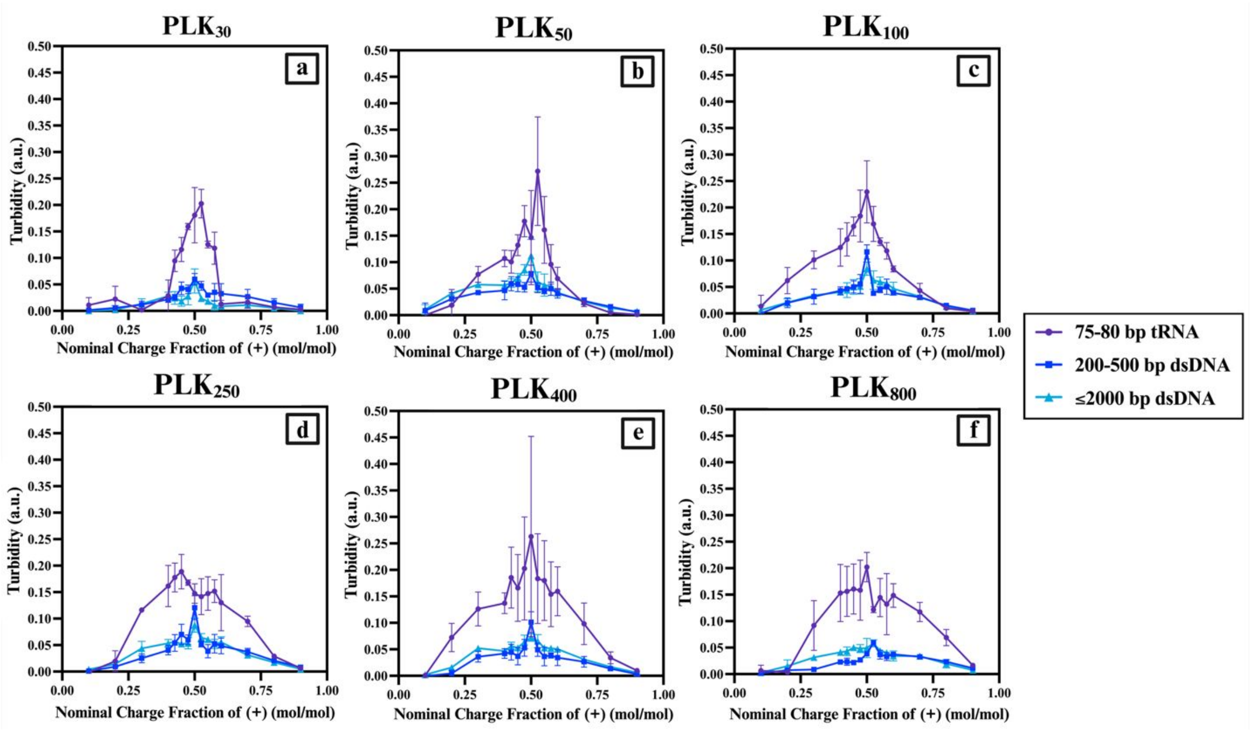

Figure S3. Turbidity (a.u.) versus nominal charge fraction of (+) (mol/mol) for all nucleic acid types for each polymer length, highlighting the effects of nucleic acid type and structure. tRNA is marked with purple triangles, 200-500 bp dsDNA is marked with light blue diamonds, and ≤2000 bp dsDNA is marked with dark blue squares. **(a)** PLK_{30} complexes, **(b)** PLK_{50} complexes, **(c)** PLK_{100} complexes, **(d)** PLK_{250} complexes, **(e)** PLK_{400} complexes, and **(f)** PLK_{800} complexes.

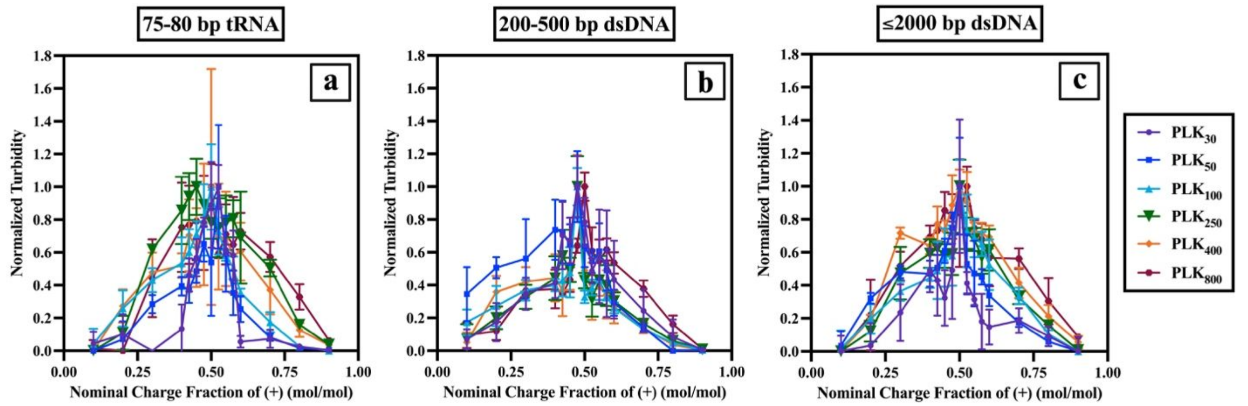

Figure S4. Normalized turbidity versus nominal charge fraction of (+) (mol/mol) for all polymer lengths, organized based on each nucleic acid type, highlighting the effects of polymer length. **(a)** 75-80 bp tRNA complexed with all PLK lengths, **(b)** 200-500 bp dsDNA complexed with all PLK lengths, and **(c)** ≤2000 bp dsDNA complexed with all PLK lengths. PLK_{30} is marked with purple spheres, PLK_{50} with dark blue squares, PLK_{100} with light blue upright triangles, PLK_{250} with green upside-down triangles, PLK_{400} with orange diamonds, and PLK_{800} with maroon hexagons.

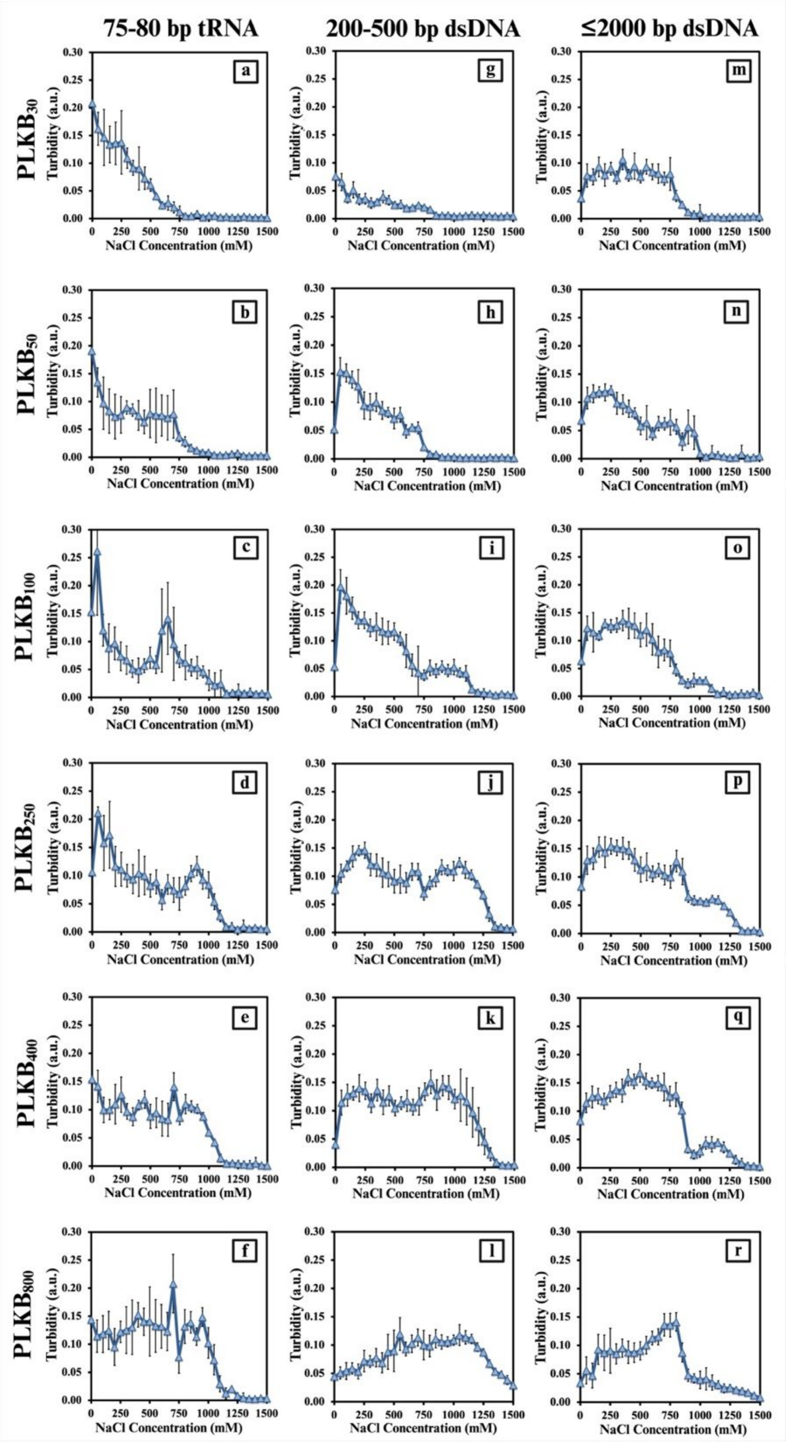

Figure S5. Turbidity versus NaCl concentration measurements for all systems at $f_{+} = 0.5$. Turbidity versus NaCl concentration curves for complexation of PLK$_{30}$, PLK$_{50}$, PLK$_{100}$, PLK$_{250}$, PLK$_{400}$, and PLK$_{800}$ with **(a-f)** 75-80 bp tRNA, **(g-l)** 200-500 bp dsDNA, and **(m-r)** ≤2000 bp dsDNA are shown.

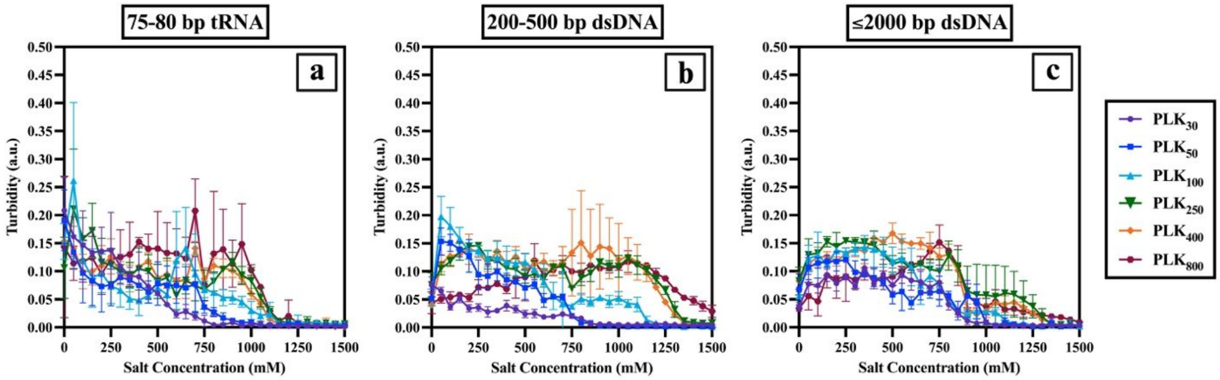

Figure S6. Turbidity (a.u.) versus NaCl concentration (mM) for all polymer lengths, organized based on each nucleic acid type, highlighting the effects of polymer length at $f_+ = 0.5$. **(a)** 75-80 bp tRNA complexed with all PLK lengths, **(b)** 200-500 bp dsDNA complexed with all PLK lengths, and **(c)** ≤2000 bp dsDNA complexed with all PLK lengths. PLK_{30} is marked with purple spheres, PLK_{50} with dark blue squares, PLK_{100} with light blue upright triangles, PLK_{250} with green upside-down triangles, PLK_{400} with orange diamonds, and PLK_{800} with maroon hexagons.

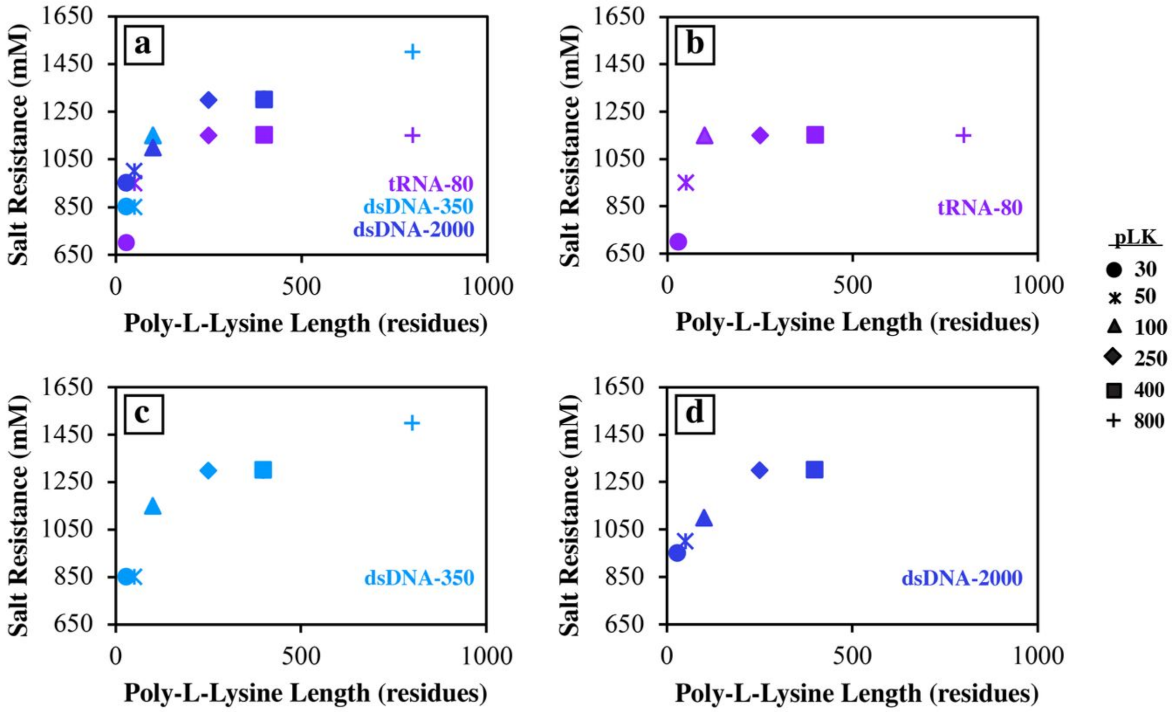

Figure S7. Salt (NaCl) resistance values as a function of poly(L-lysine) chain length (symbols) for **(a)** all three nucleic acids, **(b)** tRNA (75-80 bp, purple), **(c)** dsDNA (200-500 bp, light blue), and **(d)** dsDNA (≤2000 bp, dark blue). Note, for calculations, the rounded average for 75-80 bp tRNA and 200-500 bp dsDNA was used. The longest length (2000) was used for ≤2000 bp dsDNA.

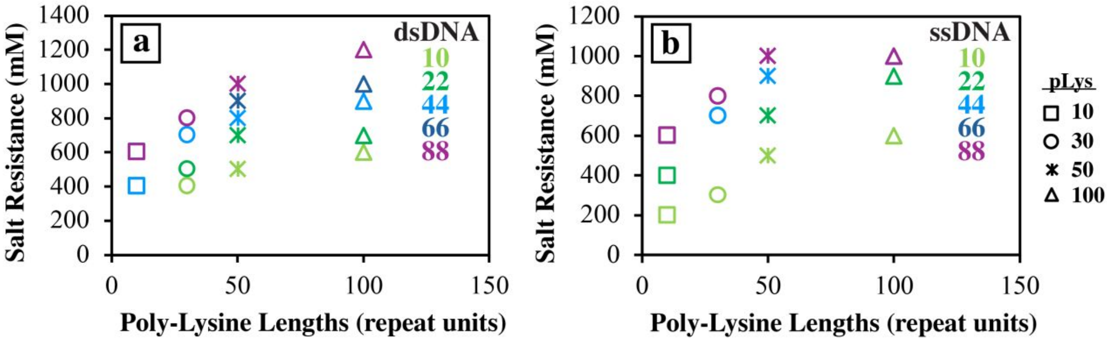

Figure S8. Short **(a)** dsDNA and **(b)** ssDNA of nt lengths 10 (light green), 22 (dark green), 44 (light blue), 66 (dark blue), and 88 (magenta) complexed with poly-lysine of aa lengths 10 (squares), 30 (circles), 50 (stars), and 100 (triangles). Data are arranged by poly-lysine length; all data from Vieregg et al.[5]

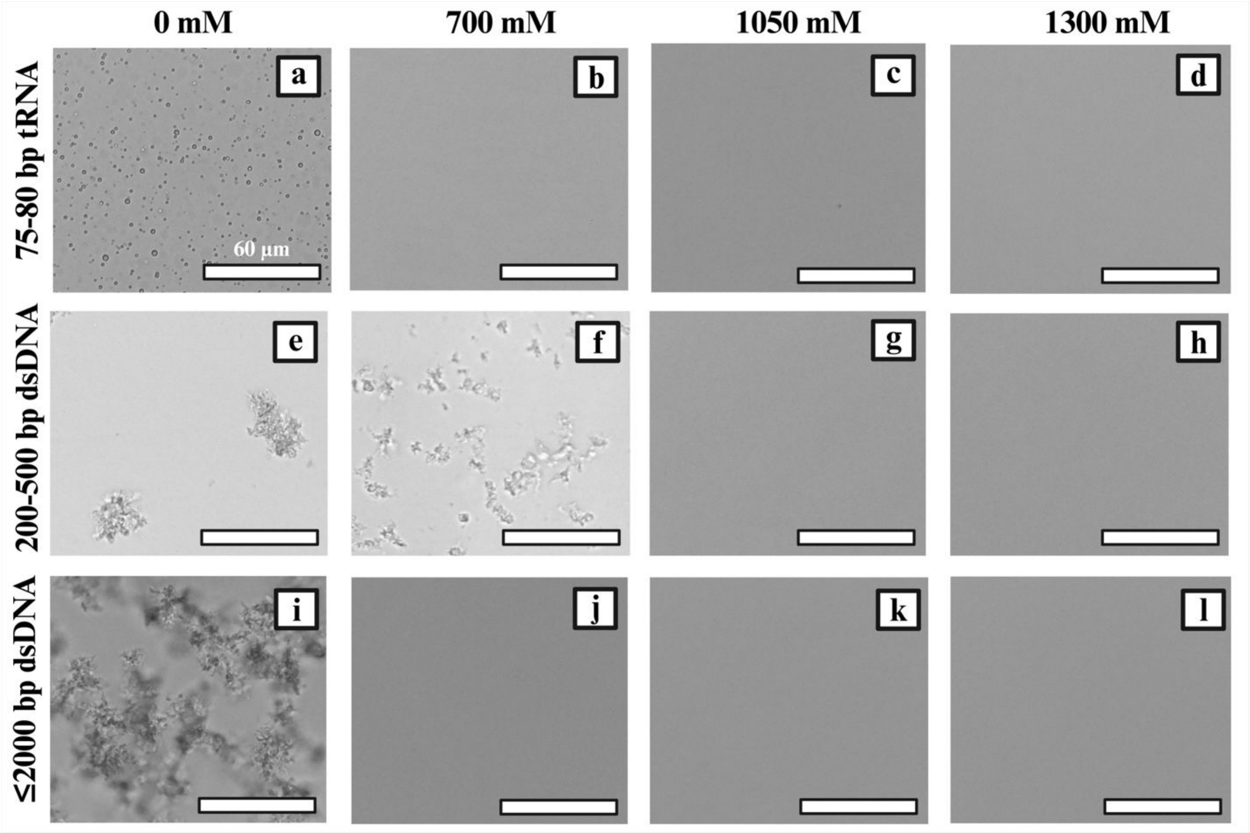

Figure S9. Micrographs of nucleic acids in complexation with PLK_{30} show changes in morphology as the concentration of added NaCl is increased. The shifts and transitions in the phase separation and morphology of PLK_{30} complexes with **(a-d)** 75-80 bp tRNA, **(e-h)** 200-500 bp dsDNA, and **(i-l)** ≤2000 bp dsDNA at 0 mM, 700 mM, 1050 mM, and 1300 mM NaCl concentrations, respectively, are shown.

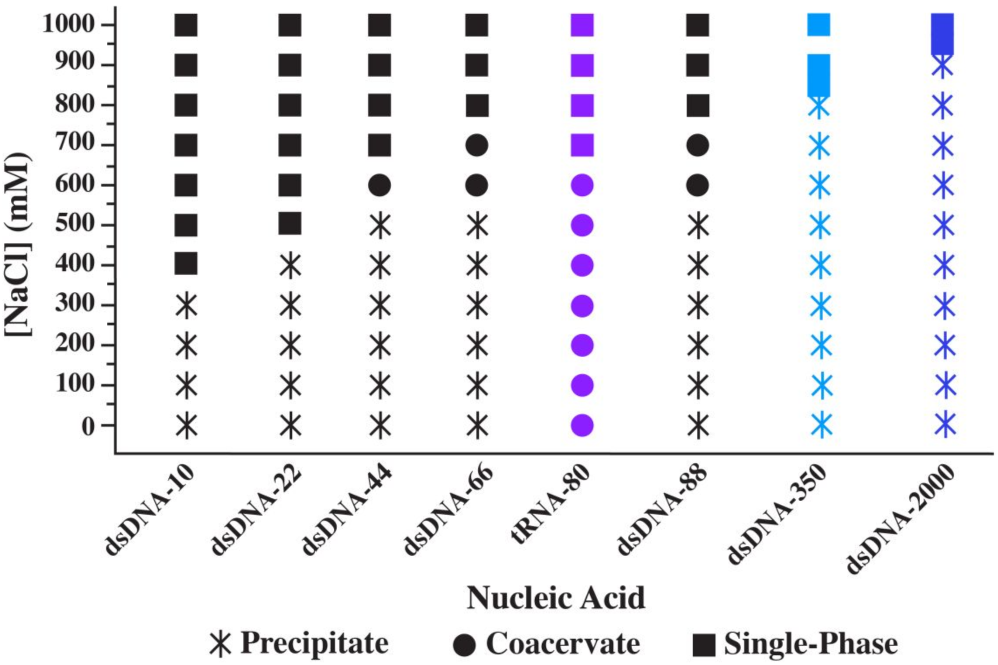

Figure S10. Phase diagram of the various nucleic acids complexed with poly-lysine of N=30. Black data are compiled from Vieregg et al.,[5] and colored data are from this work with 75-80 bp tRNA in purple, 250-500 bp dsDNA in light blue, and ≤ 2000 dsDNA in dark blue. Stars represent precipitates, circles coacervates, and squares single-phase solution.

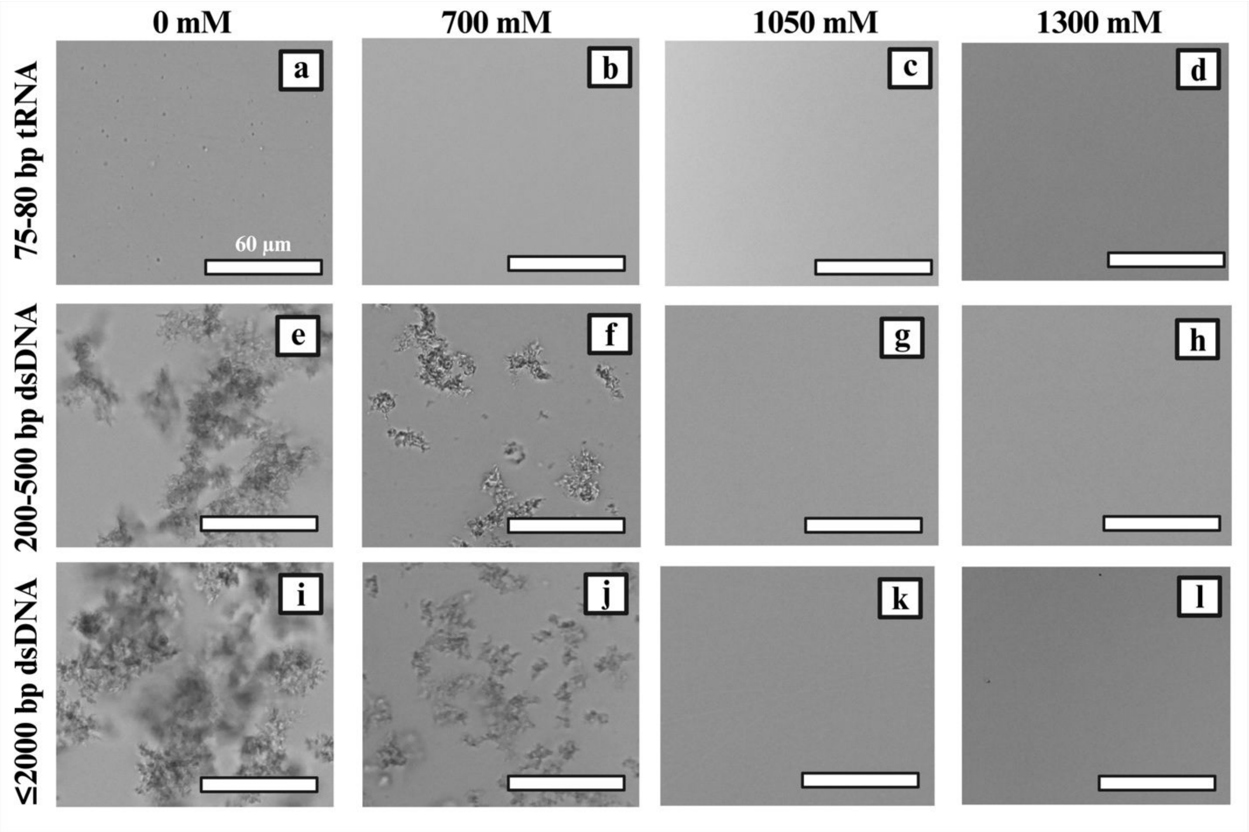

Figure S11. Micrographs of nucleic acids in complexation with PLK_{50} show changes in morphology as the concentration of added NaCl is increased. The shifts and transitions in the phase separation and morphology of PLK_{50} complexes with **(a-d)** 75-80 bp tRNA, **(e-h)** 200-500 bp dsDNA, and **(i-l)** ≤2000 bp dsDNA at 0 mM, 700 mM, 1050 mM, and 1300 mM NaCl concentrations, respectively, are shown.

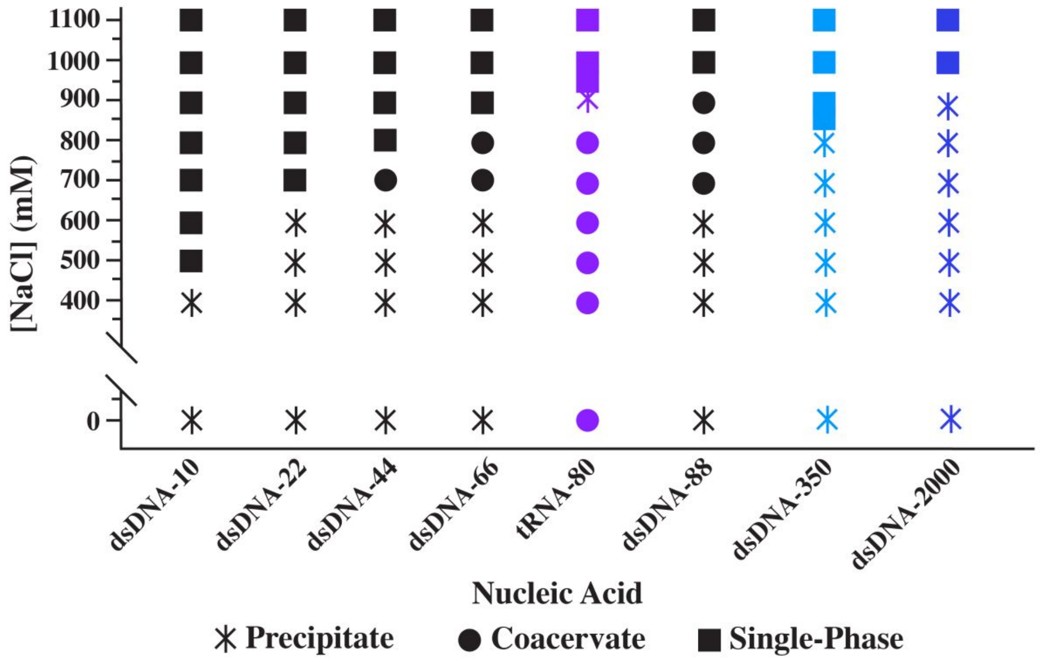

Figure S12. Phase diagram of the various nucleic acids complexed with poly-lysine of N=50. Black data are compiled from Vieregg et al.,[5] and colored data are from this work with 75-80 bp tRNA in purple, 250-500 bp dsDNA in light blue, and ≤ 2000 dsDNA in dark blue. Stars represent precipitates, circles coacervates, and squares single-phase solution.

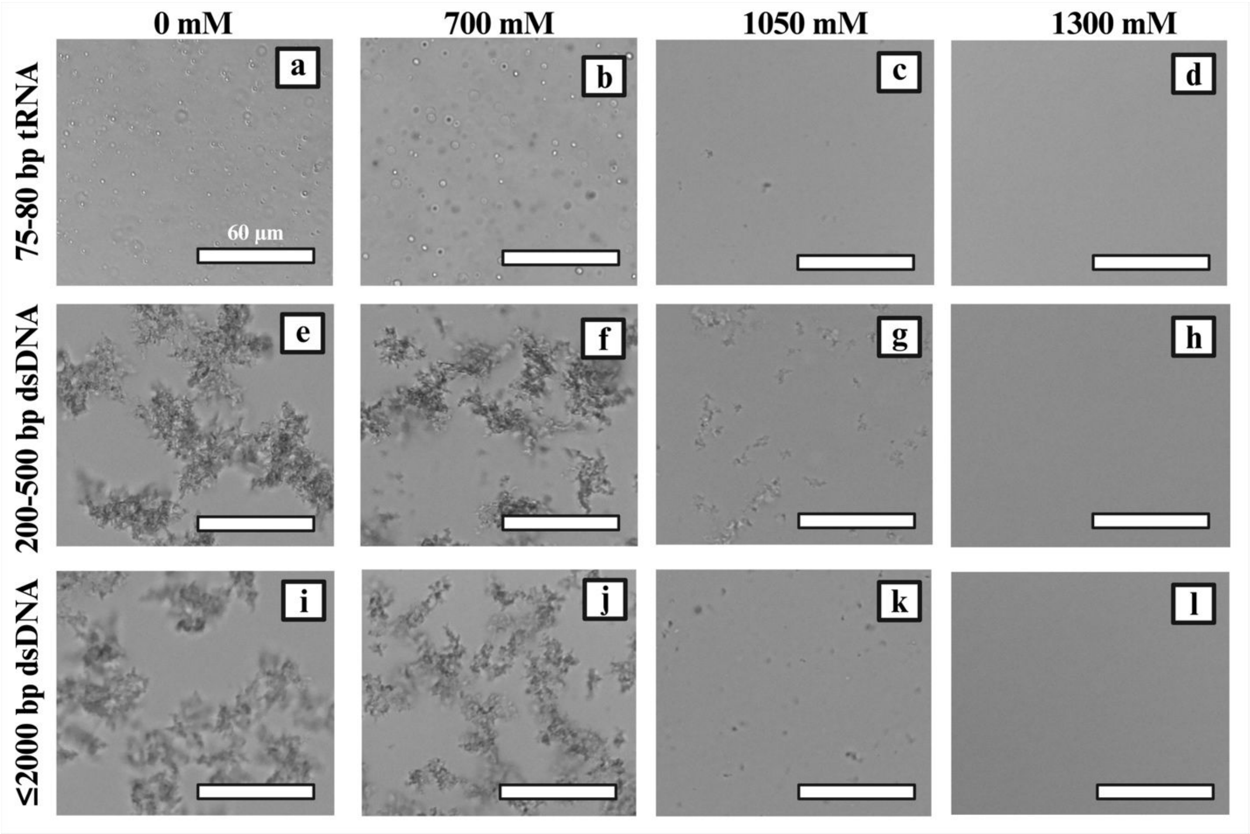

Figure S13. Micrographs of nucleic acids in complexation with PLK_{100} show changes in morphology as the concentration of added NaCl is increased. The shifts and transitions in the phase separation and morphology of PLK_{100} complexes with **(a-d)** 75-80 bp tRNA**, (e-h)** 200-500 bp dsDNA, and **(i-l)** ≤2000 bp dsDNA at 0 mM, 700 mM, 1050 mM, and 1300 mM NaCl concentrations, respectively, are shown.

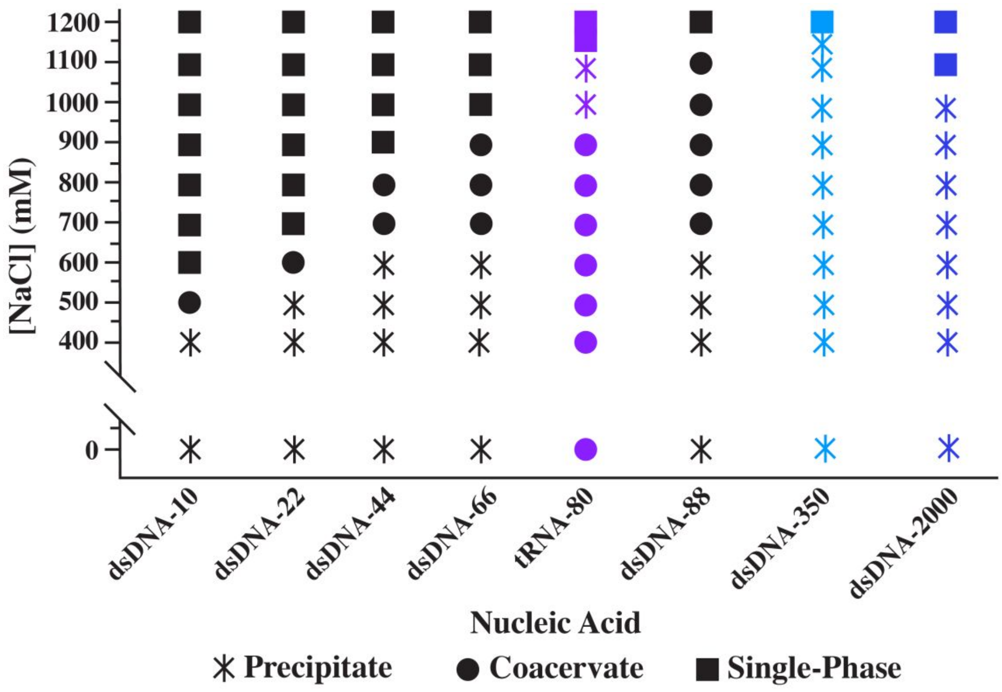

Figure S14. Phase diagram of the various nucleic acids complexed with poly-lysine of N=100. Black data are compiled from Vieregg et al.,[5] and colored data are from this work with 75-80 bp tRNA in purple, 250-500 bp dsDNA in light blue, and ≤ 2000 dsDNA in dark blue. Stars represent precipitates, circles coacervates, and squares single-phase solution.

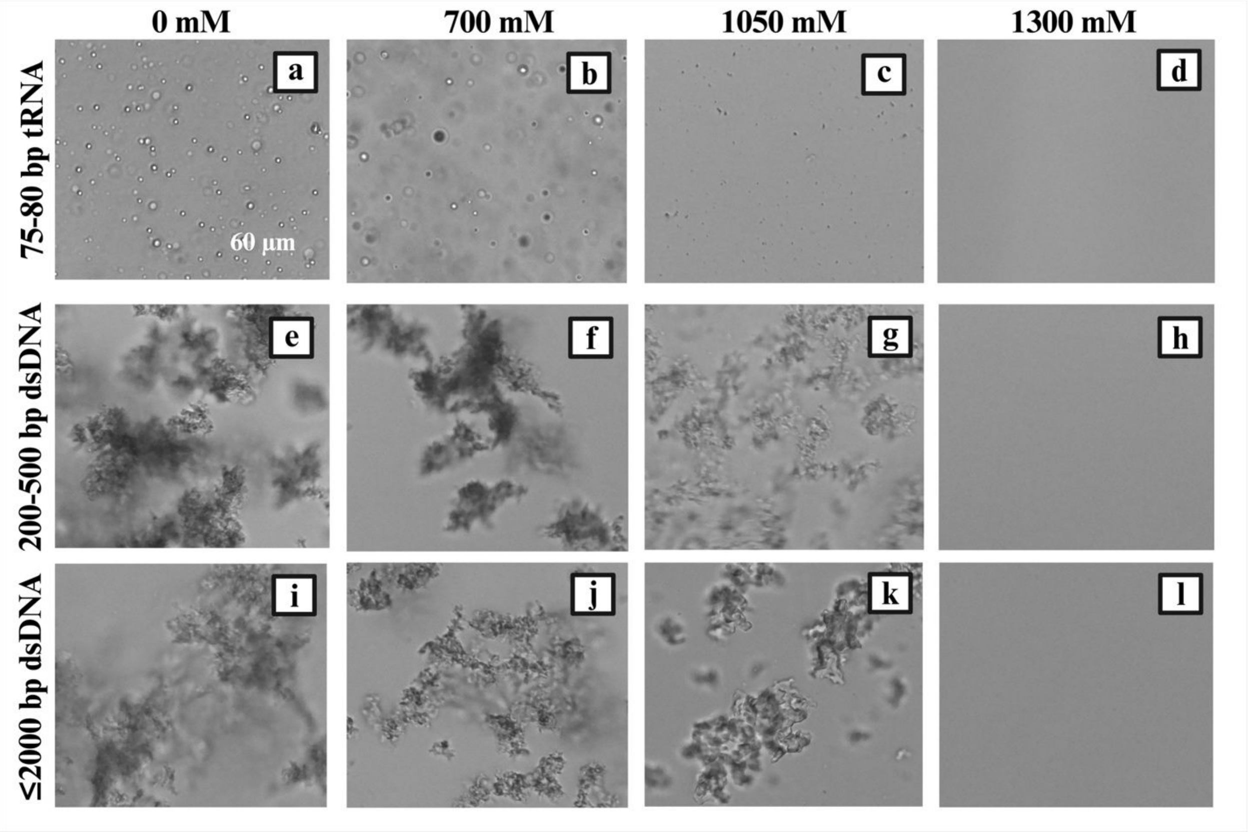

Figure S15. Micrographs of nucleic acids in complexation with PLK_{400} show changes in morphology as the concentration of added NaCl is increased. The shifts and transitions in the phase separation and morphology of PLK_{400} complexes with **(a-d)** 75-80 bp tRNA**, (e-h)** 200-500 bp dsDNA, and **(i-l)** ≤2000 bp dsDNA at 0 mM, 700 mM, 1050 mM, and 1300 mM NaCl concentrations, respectively, are shown.

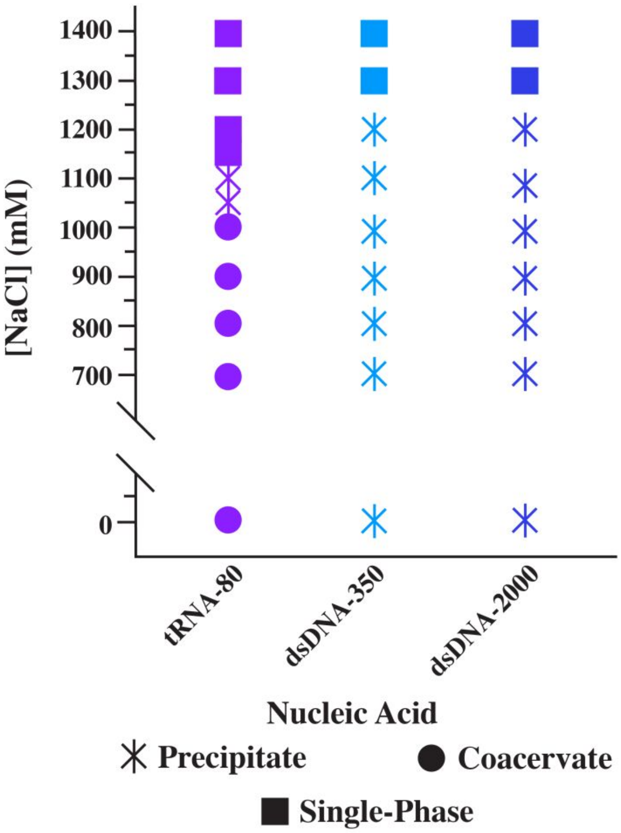

Figure S16. Phase diagram of the various nucleic acids complexed with poly-lysine of N=400. Data are from this work with 75-80 bp tRNA in purple, 250-500 bp dsDNA in light blue, and $\leq$ 2000 dsDNA in dark blue. Stars represent precipitates, circles coacervates, and squares single-phase solution.

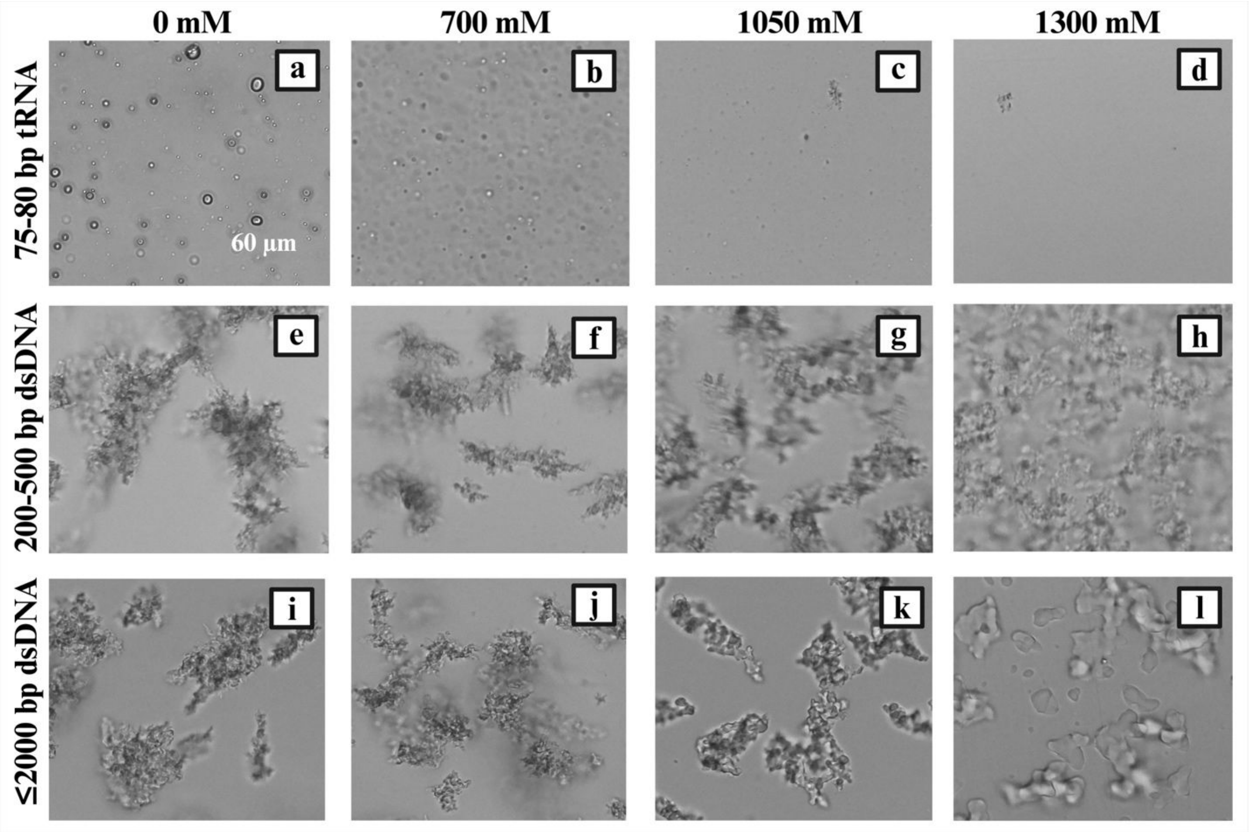

Figure S17. Micrographs of nucleic acids in complexation with PLK_{800} show changes in morphology as the concentration of added NaCl is increased. The shifts and transitions in the phase separation and morphology of PLK_{800} complexes with **(a-d)** 75-80 bp tRNA, **(e-h)** 200-500 bp dsDNA, and **(i-l)** ≤2000 bp dsDNA at 0 mM, 700 mM, 1050 mM, and 1300 mM NaCl concentrations, respectively, are shown.

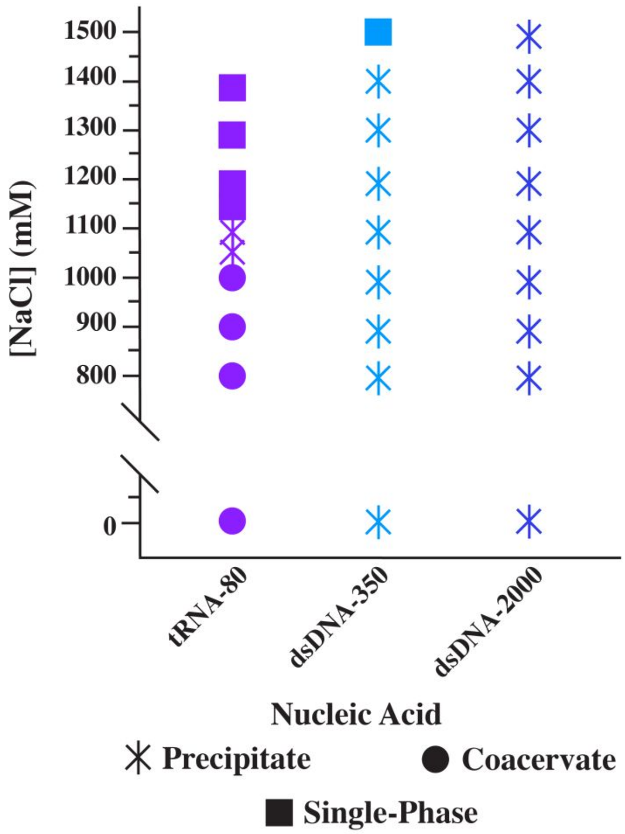

Figure S18. Phase diagram of the various nucleic acids complexed with poly-lysine of N=800. Data are from this work with 75-80 bp tRNA in purple, 250-500 bp dsDNA in light blue, and $\leq$ 2000 dsDNA in dark blue. Stars represent precipitates, circles coacervates, and squares single-phase solution.

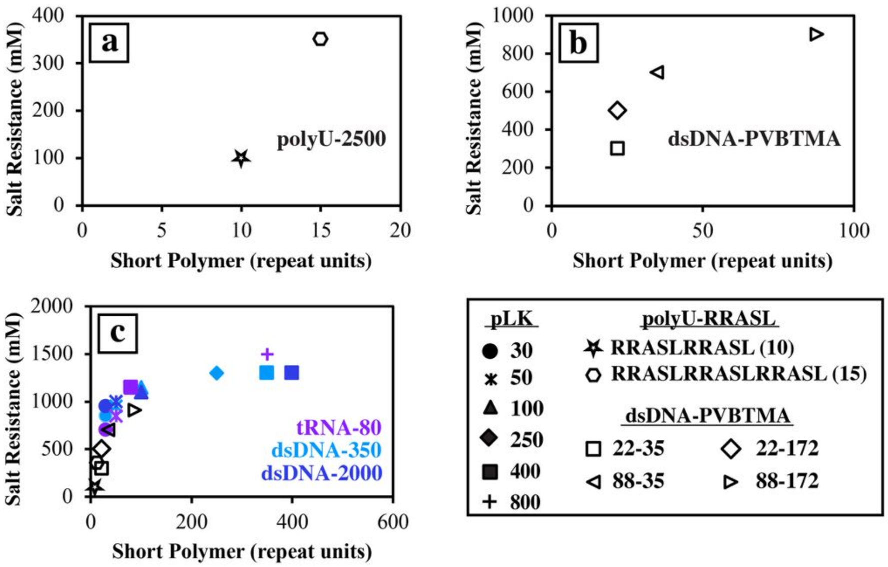

Figure S19. Salt resistance data **(a)** from Aumiller et al.[6] for poly(uracil) with average length of 2500 nt (polyU-2500) complexed with RRASL-repeat peptides of 10 (star) and 15 aa (hexagon) long, **(b)** from Marras et al.[7] for dsDNA of lengths 22 and 88 nt complexed with poly((vinylbenzyl) trimethylammonium) (PVBTMA) of lengths 35 and 172 aa, and **(c)** our results compared to these nucleic acid-polycation systems.

Table S2. Critical salt concentration (CSC) from literature sources for NaCl. The molecular weight of poly(uracil) is 600-1,000 kDa (~1850-3080 nt); the average length was used in constructing the table below.

Polyanion	Length	Polycation	Length	CSC (mM)	Reference
Poly(Uracil)	2500	RRASLRRASL	10	100	6
Poly(Uracil)	2500	RRASLRRASLRRASL	15	350	6
dsDNA	22	PVBTMA	35	300	7
dsDNA	88	PVBTMA	35	700	7
dsDNA	22	PVBTMA	172	500	7
dsDNA	88	PVBTMA	172	900	7

Table S3. Transition salt concentration (TSC) and critical salt concentration (CSC) from Vieregg et al.[5] for short dsDNA and ssDNA with poly(lysine) in NaCl.

Polyanion	Length (nt)	Polycation	Length (aa)	TSC (mM)	CSC (mM)
dsDNA	10	pLys	10	-	400
dsDNA	22	pLys	10	-	400
dsDNA	44	pLys	10	-	400
dsDNA	66	pLys	10	-	600
dsDNA	88	pLys	10	-	600
dsDNA	10	pLys	30	-	400
dsDNA	22	pLys	30	-	500
dsDNA	44	pLys	30	600	700
dsDNA	66	pLys	30	600	800
dsDNA	88	pLys	30	600	800
dsDNA	10	pLys	50	-	500
dsDNA	22	pLys	50	-	700
dsDNA	44	pLys	50	700	800
dsDNA	66	pLys	50	700	900
dsDNA	88	pLys	50	700	1000
dsDNA	10	pLys	100	500	600
ssDNA	22	pLys	100	600	700
ssDNA	44	pLys	100	700	900
dsDNA	66	pLys	100	700	1000
dsDNA	88	pLys	100	700	1200
ssDNA	10	pLys	10	-	200
ssDNA	22	pLys	10	-	400
ssDNA	44	pLys	10	-	600
ssDNA	66	pLys	10	-	600
ssDNA	88	pLys	10	-	600
ssDNA	10	pLys	30	-	300
ssDNA	22	pLys	30	-	700
ssDNA	44	pLys	30	-	700
ssDNA	66	pLys	30	-	800
ssDNA	88	pLys	30	-	800
ssDNA	10	pLys	50	-	500
ssDNA	22	pLys	50	-	700
ssDNA	44	pLys	50	-	900
ssDNA	66	pLys	50	-	1000
ssDNA	88	pLys	50	-	1000
ssDNA	10	pLys	100	-	600
ssDNA	22	pLys	100	-	900
ssDNA	44	pLys	100	-	1000
ssDNA	66	pLys	100	-	1000
ssDNA	88	pLys	100	-	1000

References

(1) Panwar, B.; Raghava, G. P. S. Prediction of Uridine Modifications in TRNA Sequences. *BMC Bioinformatics* **2014**, *15*, 326. https://doi.org/10.1186/1471-2105-15-326.

(2) *DNA and RNA Molecular Weights and Conversions.* https://www.thermofisher.com/us/en/home/references/ambion-tech-support/rna-tools-and-calculators/dna-and-rna-molecular-weights-and-conversions.html (accessed 2025-09-19).

(3) Chan, P.; Lowe, T. GtRNAdb: A Database of Transfer RNA Genes Detected in Genomic Sequence. *Nucleic Acids Res.* **2009**, *37(Database issue)*, D93–D97.

(4) Chan, P. P.; Lowe, T. M. GtRNAdb 2.0: An Expanded Database of Transfer RNA Genes Identified in Complete and Draft Genomes. *Nucleic Acids Res.* **2016**, *44(Database issue)*, D184–D189.

(5) Vieregg, J. R.; Lueckheide, M.; Marciel, A. B.; Leon, L.; Bologna, A. J.; Rivera, J. R.; Tirrell, M. V. Oligonucleotide-Peptide Complexes: Phase Control by Hybridization. *J. Am. Chem. Soc.* **2018**, *140* (5), 1632–1638. https://doi.org/10.1021/jacs.7b03567.

(6) Aumiller, W. M.; Keating, C. D. Phosphorylation-Mediated RNA/Peptide Complex Coacervation as a Model for Intracellular Liquid Organelles. *Nat. Chem.* **2016**, *8* (2), 129–137. https://doi.org/10.1038/nchem.2414.

(7) Marras, A. E.; Vieregg, J. R.; Ting, J. M.; Rubien, J. D.; Tirrell, M. V. Polyelectrolyte Complexation of Oligonucleotides by Charged Hydrophobic-Neutral Hydrophilic Block Copolymers. *Polymers (Basel).* **2019**, *11* (1). https://doi.org/10.3390/polym11010083.
